# GHz Ultrasonic Chip-Scale Device Induces Ion Channel Stimulation in Human Neural Cells

**DOI:** 10.1038/s41598-020-58133-0

**Published:** 2020-02-20

**Authors:** Priya S. Balasubramanian, Ankur Singh, Chris Xu, Amit Lal

**Affiliations:** 1 School of Electrical and Computer Engineering, Cornell University, Ithaca, 14853 NY USA; 2 Sibley School of Mechanical and Aerospace Engineering, Cornell University, Ithaca, 14853 NY USA; 3 Meinig School of Biomedical Engineering, Cornell University, Ithaca, 14853 NY USA; 4 School of Applied and Engineering Physics, Cornell University, Ithaca, 14853 NY USA

**Keywords:** Biophysics, Biomedical engineering

## Abstract

Emergent trends in the device development for neural prosthetics have focused on establishing stimulus localization, improving longevity through immune compatibility, reducing energy re-quirements, and embedding active control in the devices. Ultrasound stimulation can single-handedly address several of these challenges. Ultrasonic stimulus of neurons has been studied extensively from 100 kHz to 10 MHz, with high penetration but less localization. In this paper, a chip-scale device consisting of piezoelectric Aluminum Nitride ultrasonic transducers was engineered to deliver gigahertz (GHz) ultrasonic stimulus to the human neural cells. These devices provide a path towards complementary metal oxide semiconductor (CMOS) integration towards fully controllable neural devices. At GHz frequencies, ultrasonic wavelengths in water are a few microns and have an absorption depth of 10–20 *µm*. This confinement of energy can be used to control stimulation volume within a single neuron. This paper is the first proof-of-concept study to demonstrate that GHz ultrasound can stimulate neurons *in vitro*. By utilizing optical calcium imaging, which records calcium ion flux indicating occurrence of an action potential, this paper demonstrates that an application of a nontoxic dosage of GHz ultrasonic waves $(\ge 0.05\frac{W}{c{m}^{2}})$ caused an average normalized fluorescence intensity recordings >1.40 for the calcium transients. Electrical effects due to chip-scale ultrasound delivery was discounted as the sole mechanism in stimulation, with effects tested at *α* = 0.01 statistical significance amongst all intensities and con-trol groups. Ionic transients recorded optically were confirmed to be mediated by ion channels and experimental data suggests an insignificant thermal contributions to stimulation, with a predicted increase of 0.03 ^*o*^*C* for $1.2\frac{W}{c{m}^{2}}\cdot$ This paper paves the experimental framework to further explore chip-scale axon and neuron specific neural stimulation, with future applications in neural prosthetics, chip scale neural engineering, and extensions to different tissue and cell types.

## Introduction

Neural interfaces can be used to implement human assistive devices to improve the quality of life of patients by restoring nerve signal pathways, improving communications, and motor functions^1^. These devices can be used for individuals that have undergone stroke, spinal cord injuries, brain disease related damage, peripheral nerve damage for which there is significant clinical burden. For example, there are an estimated 17,700 new cases of spinal cord injuries per year^2^. Any technology used to for neuron activation should ideally be long-lasting, preferably with lifelong performance for the patient. Prosthetic microsystems range in size from nanometer to centimeter scale, and include optical probes for optogenetic methods, electrode arrays (micro and nanowire or planar electrodes), and ultrasonic transducers^3–7^. However, major challenges exist in the development of long-term stable neural prosthetics, including (1) controlled neuron specific excitation within biologically unmodified tissue, (2) interference from scar tissue, and (3) invasiveness of current techniques are outstanding problems^8–10^.

Ultrasonic neuromodulation offers key advantages over other existing stimulatory methods such as optogenetics and electrical stimulation^3,4^. Although optogenetics can achieve high spatial resolution and localization, on the order of *λ*_*stimulation*_, it does so at the expense of genetic modification of cells^5–7^. Optogenetic methods can further be limited by the requirement of wavelength specificity and difficulty of light guidance to specific neurons, and associated phototoxicity^5,6^. Electrical neurostimulation, such as deep brain stimulation (DBS) and cortical stimulation, are widely applied techniques for neuromodulation in neurological disorders with high spatial resolution^11,12^. However, the approach is invasive and the electrodes, which are often made of micro needle contact wires and conductive thin film tracks on silicon micro-machined substrates, can displace and damage tissue, and may elicit an immune response. The inflammatory response to the electrode surface results in electrically insulating scar tissue layers over the electrodes, therefore preventing reliable long-term neural interfaces, neuronal cell death, and eventual loss of electrode function^13–15^. Ultrasound waves can penetrate through scar tissue adjacent to the device, reaching the neurons for stimulation. In addition, ultrasonic transducer integrated with and controlled by CMOS (Complementary Metal Oxide Semiconductor) electronics can implement closed loop systems that can feedback regulate delivered ultrasonic power, and also communicate to the external electronics through RF or ultrasonic communication links. The GHz ultrasonic excitation, owing to very small wavelengths and short penetration distance potentially allows for axon-level excitation without invasive penetration into a nerve, distinguishing GHz ultrasonics from lower frequency ultrasonics.

**Figure 1:**
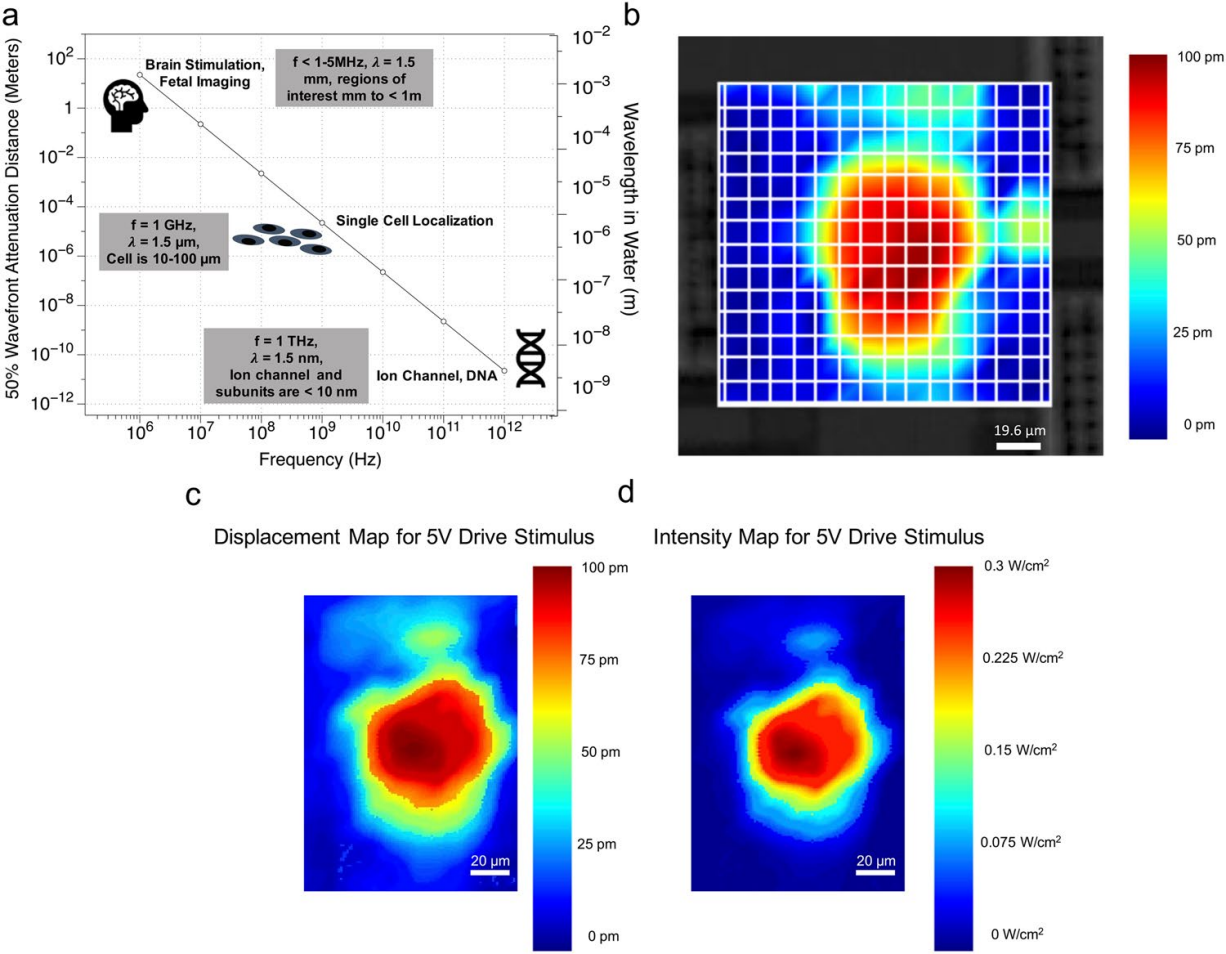
GHz localization and Ultrasound Field Confinement Measurement. (**a**) GHz ultrasonics, 10^9^ Hz, is capable of axial localization to the single cell level, enabled by both wavelength reduction and increase in attentuation in tissue and water. (**b**) The displacement (in picometers), measured with optical interferometry. The displacement was generated by a 70 *µ*m AlN piezoelectric transducer on the opposite side of a 750 *µm* thick silicon wafer. The beam energy is confined to an area of 140 × 140 *µm*, which can contain several neurons. (**c**) Another displacement vibrometer recording displayed without grid bar and background device display. (**d**) Data from Fig. 1c plotted as power density, in *W/cm*^2^ adjusted to the power in water corresponding to displacement in air.

Ultrasonic neuromodulation are typically conducted with transducers generating waves from 100 kHz to 10 MHz^16–20^. At these frequencies, the longer wavelengths and larger focal volumes can excite large ensemble of neurons, and lower propagation loss allows for deeper tissue penetration. Ultrasonic wavelengths at 1 GHz are 5–10 *µm* in solids and 1.5 *µm* in liquids, corresponding to speeds of sounds of ~5000–10,000 m/s in solids, and ~1500 m/s in fluids and tissue. Following from basic ultrasonic wave propagation principles, GHz ultrasonic waves can be focused to approximately one wavelength, although typical spot sizes are several wavelengths in diameter and fine features can be resolved. Figure 1a shows the potential for localization based on scaling of the wavelength and absorption. Figure 1b demonstrates the spatial extent of an ultrasonic beam at GHz frequencies achieved in this paper for a far-field diffraction of a GHz wavefront from a 70 *µm* square AlN thin film ultrasound transducer. Figure 1c,d shows data from a second repeated scan with information on the confinement of the displacement map for the air interface and the power density for the water interface respectively. Concurrently, the absorption depth at GHz frequencies is also in the tens of microns range owing to high absorption in liquids and tissue^21^. Recent research on GHz photoacoustic imaging provides exemplary data on the high spatial resolution at GHz ultrasonics^22^. Surface Acoustic Microscopy at GHz frequencies has also been used to image single cells^23^. Ultrasonic beam focused to a wavelength and beam absorption within 10 s of microns provides the unique opportunity to confine stimulus to volumes of 10 *µm* on each side (Fig. 1). Since neurons are in 10–30 *µm* in size^24^, GHz ultrasonic waves have the potential to be used for stimulation of single neurons. Since the focal volumes can be smaller than the neural volume, focused beams within a neuron could be controlled to excite sub-cellular components of neurons. The GHz ultrasound stimulation technique has the potential for targeted, localized therapeutic delivery of stimulation to highly specific, narrowly focused neural cells and closed-loop brain-machine interfaces. In addition, the study of high-frequency ultrasonic stimulation is interesting as new insights on ultrasonic interaction with various sub-cellular components might elucidate mechanisms of ultrasonic neuromodulation better than then the lower frequency neuromodulation.

Supplementary Table S4 shows a summary of stimulus modalities, including kHzMHz ultrasound, optogenetic optical stimulation, and electrical stimulation. Each section includes relevant recent work in the area, along with the resultant effects of stimulation utilizing a particular modality. For all techniques, both stimulation and suppression of neural activity can be observed depending on the tissue type and stimulation modality used. The reasoning for this can range from the mechanical effects to a lack of localization causing unintentional effects. The major contributor in utility of these techniques lies in the following – (1) localization and penetration (2) toxicity and dosage effects (3) longevity of delivery modality, if implantable. When evaluating methods, kHz-MHz ultrasonics cannot localize well due to longer wavelengths. Electrode arrays suffer with limited lifetime owing to scar tissue formation over the electrodes. Longevity of the signal is also a problem with optogenetics given the transient nature of gene transfection and cell turnover rate. Additionally, optogenetics can have adverse effects due to phototoxicity and transfection toxicities that lower cell viability, hampering long-term viable clinical applications. Hence, optogenetics has remained more predominantly a research tool. This provides a very clear rationale for localized ultrasonic stimulus – the dependency of stimulation effect on tissue type and localization provides adequate need for delivery of energy to smaller regions to not only understand the effects of ultrasound on tissue but also more precisely utilize known effects of ultrasound on tissue. Furthermore, precision of localization of delivery lends way to implantable devices that have further ability to target tissue without creating more global, tissue scale effects. Additionally, toxicity and risks associated with ultrasonic methods are lower and well characterized.

Recent work has demonstrated that piezoelectric AlN (Aluminum Nitride) transducers can be integrated with CMOS (Complementary Metal-Oxide Semiconductor) wafers as a low-cost post-CMOS process^25^. Solidly mounted AlN transducers onto CMOS wafers has further enabled high resolution imaging for fingerprint sensing^26^, ultrasonic delay line memory^27^, temperature sensing, and ultrasonic delay lines^28^. CMOS integrated GHz ultrasonic transducers could enable reliable, long-term stable neurostimulation, then highly integrated systems consisting of signal and data processing and RF transmission on the same chip are possible owing to the complexity of circuitry that can be integrated using CMOS technology. On top of this, this GHz technology enables high energy stimulation to cells at a given drive voltage due to the high frequency stimulus.

In the current study, one of the first documented effects of GHz stimulation in human neural cells is demonstrated. This result paves a path towards localized, CMOS integrated ultrasonic stimulation and sensing neural technologies. The results from this paper create a foundation to further develop GHz ultrasonic chip-scale devices for use as life-long implantable neural prosthetics. Neural stimulation performed in this paper is driven by AlN ultrasound transducers fabricated on silicon substrates. The transducers generate 1.47 GHz ultrasonic waves into the silicon die. Wave-fronts emanating from the silicon wafer side, the side opposite to the transducers, is coupled into the water and into the *in vitro* tissue prep. At 1.47 GHz and drive amplitudes 0–5 *V*_*peak*_ across the AlN transducer generate peak-to-peak surface displacements ≤100 pm. The estimated ultrasonic intensity in water at the water/silicon wafer interfaces is $\le 0.3\,W/c{m}^{2}(\langle I\rangle =\frac{1}{2}\rho c{(\omega {u}_{peak})}^{2})$. This intensity is within the range of ultrasonic intensities previously determined to be under the toxicity threshold to elicit neuromodulation^20,29,30^. In the current work, transducers affected more than one cell due to larger transducer size, inter-neuron connectivity, and secondary effects such as acoustic streaming induced flow. Nevertheless, this study provides direction towards single cell isolation with further device, and beam development. In addition, given the attenuation length axial resolution is confined to ~10–20 *µm*, hypothetically influencing a maximum of a few layers of neurons in tissue structure. GHz ultrasonics, due to the unique physics and energetics, can alone stimulate ion channels mechanically, without necessary assistance from thermal or electrical stimulation, as this study will detail.

## Methods

### Piezoelectric AlN transducers

**Figure 2:**
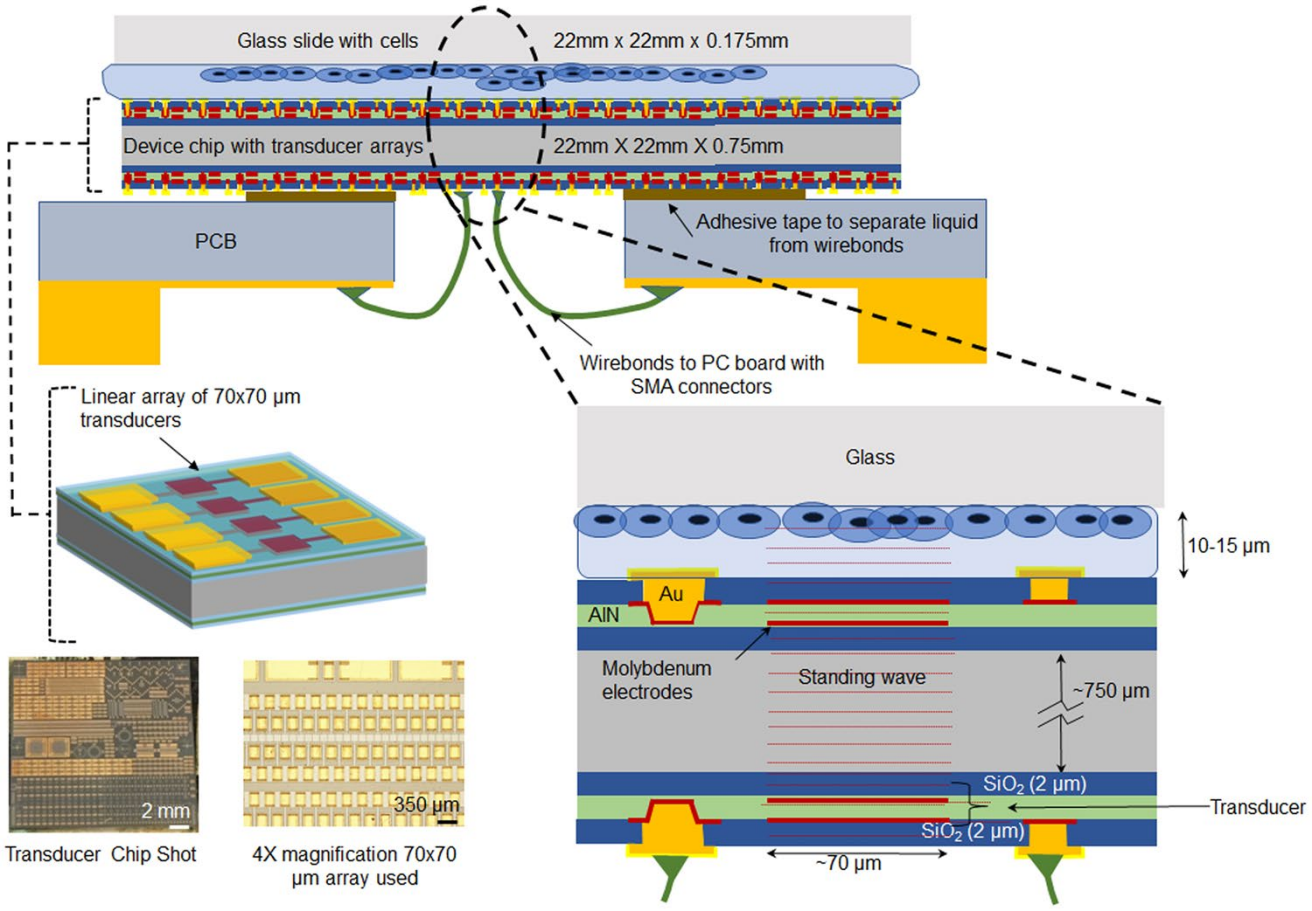
Sketch of ultrasonic transducer chip and packaging used for neural stimulation studies. (top) The AlN transducer chip is adhesively attached to an orifice in a PC board. Wire bonds are used to connect the transducer electrodes to PCB traces connected to SMA RF connectors. A glass cover slip with cell grown on the glass is placed on top of the chip with a thin media layer in between the glass and the chip. (bottom left) The silicon die is 22 mm × 22 mm with AlN transducers are either on one side or both sides of the chip. Gold electrodes on bond pads are used to connect to the PC board via wire bonds. Chip shot is taken after use with neurons, and no metal delamination or damage is observed. (bottom right) Waves generated from the bottom transducer setup a standing wave pattern through the die thickness affecting the neurons. Note that in the two-sided transducer silicon die, the electrodes for the top transducers are floating. The top transducers are aligned to the bottom transducers and are used to locate the cells in reference to the actuated transducers.

AlN transducers were fabricated on double-polished silicon wafers (725 *µm* thick). Transducers were fabricated either only one side (referred to in this paper as single-sided or electrode free surface), or on both sides of the wafer. In the two-sided transducer wafers, the front and back transducers are aligned to each other and are near identically fabricated. The two-sided transducer allows knowing the relative position of the cells to the drive transducers on the opposite side of the wafer not optically visible (Fig. 2). Although the transducers on the front-side, closer to the biological sample can be used to excite the samples, the electrical connectivity is more challenging as the wirebonds need to connect to the transducers would need to be electrically isolated from the cell sample. Furthermore, the use of backside transducers allows for through silicon ultrasonic beam forming through the silicon wafer. Since the top transducers are active piezoelectric transducers, used only for optical location identification, the voltages across them due to incident ultrasonic waves can potentially affect the cells. Hence, chips with silicon only on the cell interface side (one sided transducer fabrication with a blank silicon surface) are used as control for identifying the potential effect of in-active transducers on the cell side and decoupling possible electrical stimulation as the sole factor in stimulation.

The 2 *µm* AlN layer with a 2 *µm* oxide layer between the 725 *µm* silicon and AlN results in a thickness-mode resonance at 1.47 GHz. The transducers have a film of a 2 *µm* PECVD SiO_2_ on the top surface. Molybdenum (Mo) electrodes (0.2 *µm* thick) at the top and bottom of the AlN films are electrically driven to apply electric fields across the AlN transducers. The transducers were fabricated as 70 *µm* square transducers with a 100 *µm* pitch in a linear array. A glass coverslip with neuronal cells was transferred to the transducer array and positioned with several neurons directly over the transducer. Typically, a single transducer has ~5 cells in proximity of each other is in the field of view of the calcium transient recording. The motion produced on the water interface has a high-intensity center lobe and some secondary lobes as shown in Fig. 1b–d. This localizes the high intensity portion of the acoustic pressure field to at most a few cells during stimulation testing.

### Neural cell culture and differentiation protocol

**Figure 3:**
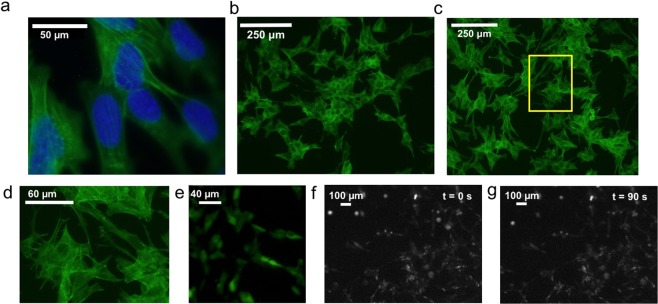
SH-SY5Y cell development and staining characterization. (**a**) F-Actin (Phalloidin) and DAPI stained 48-hr RA differentiated cells at 100×. Synapse shown in red circle. (**b**) Undifferentiated SH-SY5Y cells (F-actin Phalloidin stain). (**c**) 48 hour differentiation in RA (F-actin Phalloidin stain). (**d**) Yellow box from 3c shown at higher digital magnification e. Fluo-8 AM loaded cells. (**f**) Cells in preparation on chip for stimulation before ultrasound exposure and t = 0. (**g**) Cells on chip in preparation after ultrasound exposure at t = 90, little to no change in position and no morphological changes immediately notable with exposure time used in this study.

SH-SY5Y neuroblastoma cells (acquired from Sigma Aldrich) are differentiated using retinoic acid that rendered these cells with a more neural phenotype. This neural-like cell line was used to preserve the tissue engineering and cell differentiation applications in this particular study as opposed to using primary tissue sourced neurons or brain slices. SH-SY5Y neuroblastoma cells were seeded at a density of 15,000 cells/cm^2^onto glass slides. After an initial 48 hours that of precursor seeding and growth, a final cell surface density was observed at ~50–70% cell confluency. Subsequently, these cells were differentiated to push from epithelial to neural phenotype for another 48 hours. The retinoic acid was stored in a 5 mM DMSO stock solution, and diluted to 10 mM in partially serum deprived (2.5% FBS), glutamine enhanced (2 mM), Eagle’s Minimum Essential Medium (EMEM) medium. Culture medium was treated with Penicillin and Streptomycin (1X PenStrep from 100X stock solution). Cells were grown in and incubator at 37 °C and 5% CO_2_. The cells were then prepared by loading calcium imaging agent for calcium ion transient imaging. While the complete differentiation protocol involves several other neurotrophic factors, this protocol used RA differentiation until a time point of observable neurites, as shown in Fig. 3a–d. RA differentiation is substantiated by past work and allows for more confluent cell populations and stronger surface adhesion compared to later differentiation time points^31,32^. High cell surface density (50–70%) and strong surface adhesion are desired to preserve large sample size and prevent cell detachment from the glass cover slip due to ultrasonic forces. Further differentiation leads to low cell density and poor adhesion, requiring reseeding and surface coating, which are suboptimal for this study^31^. Hence the 48-hour stage, when clear appearance of neurites is noted, was chosen as the differentiation end point. The glass slide with seeded and growing cells was interfaced to the ultrasonic transducer chip with a gap of 10–15 um between the transducer and the sample cover slip (Fig. 2). This distance is tunable using a controlled volume of cell culture media between the glass slide and silicon chip. Experiments described in this paper are set up with 10 *µL* of cell media to control the spacing to approximately 10 *µm*, measured using the focus of an optical microscope.

### Calcium imaging loading parameters

Calcium imaging is used to obtain information on the activity of the neurons and quantify and describe calcium transients in the cell preparation that is interfaced with high frequency ultrasonic. In order to image these calcium transients, Fluo-8 AM (purchased from SantaCruz Technologies) was loaded into the cells. Fluo-8 AM reagent was stored in stock at 5 mM in Dimethyl Sulfoxide DMSO (Fisher Scientific). Cells seeded as described in the previous section onto glass slides were rinsed three times in HHBS (Hank’s Buffer with 20 mM Hepes – formulation compatible with Fluo-8AM) and incubated at 37 °C and 5% CO_2_ for 5 minutes. After incubation, an equal volume of 5 mM solution of Fluo-8 AM is placed onto the glass slide. The cells were incubated for 60 minutes with this dye-containing solution. The Fluo-8 AM working solution was then removed, the cells were rinsed three times with HHBS and again incubated with HHBS for 20 minutes at 37 °C and 5% CO_2_. Each glass cover slip was kept in the incubator until interfaced with the ultrasonic device and imaged, which occured for a maximum of 120 seconds at room temperature. The imaging was performed on a reconstructed Zeiss Axioplan 2 with a 10X air immersion objective with a Thorlabs 340M-USB fast frame rate camera. The excitation/emission filters were within Zeiss FITC filter range.

### F-actin and nuclear staining

DAPI (4^*j*^,6-diamidino-2-phenylindole) and Phalloidin staining was used to visualize the nucleus and F-actin respectively of SH-SY5Y cells. DAPI (excitation 350 nm, emission 470 nm) and Phalloidin (conjugated with Alexa-Fluor excitation 490 nm, emission 525 nm) stains were applied to the same sample due to the non-overlapping excitation and emission spectrum. Standard protocols are followed with recommended concentrations as provided by the vendor, Sigma Aldrich and Thermo Fisher Scientific.

### Eu-TTA thermal imaging loading parameters

In order to image thermal change in the cell preparation with ultrasonic stimulus, Europium (III) Thenoyltrifluoro-acetonate, or Eu-TTA was used to stain the cells. Eu-TTA was stored in DMSO at 10 mM at −20 °C. It was prepared in buffered saline solution (HBSS) at a concentration of 50 *µM* as a loading solution. Cells prepared as described on glass slides were rinsed three times with buffered saline solution. The cells were then incubated with 200 *µL* of loading solution on each slide for 30 minutes at 37 °C and 5% CO_2_. The cells were subsequently rinsed in HHBS three times again, and imaging was performed with a HHBS controlled volume interface (10 *µL*). Given loading heterogeneity, in order to obtain the best metric for temperature change with ultrasonic exposure, the temperature calibration data was obtained with the stock loading solution itself with no cells present at three temperatures (26–28 °C) corresponding to room temperature and 2 degrees higher than room temperature. This provides the most consistent measure for calibration. While there is a small shift in excitation/emission when Eu-TTA is not bound to cells, the range of the filter sets used covers subtle changes in optimal excitation/emissions (240–395 nm UV-passing excitation, 425 nm long pass dichroic, 510 nm long pass emission, from Thorlabs)^33^. Ten sets of images were obtained using the OMAX A35140U camera. The data with cells before ultrasonic stimulation was compared to immediately after ultrasonic stimulation and the intensity difference between each cell classified region (as described in the image processing section) was compared and added to the known room temperature, with the final result depicted being I_*after*_- I_*before*_ + I_26_ _°C_. This is depicted graphically in the results section. The temperature this intensity corresponds to was approximated by linear interpolation of the control temperature data. Number of ROIs is reported the results.

### Gentamicin Ca^2+^ ion channel blocker

In order to test the effect of ultrasound on the ion-channels, a control experiment was also conducted to block voltage and mechanically gated calcium ion-channels^34,35^. Gentamicin (Thermo Fisher Scientific) was prepared at 200 *µM* in HHBS solution and incubated with the cells already loaded and incubated with Fluo-8 AM after three washes with HHBS. The gentamicin loaded solution was incubated with cells for 60 minutes at 37 °C and 5% CO_2_.

### Ultrasonic beam characterization

The transducers create a wavefront that travels through the silicon wafer thickness, which is ~85 wavelengths thick. The travel distance of the wavefront is the thickness of the Silicon substrate as indicated in Fig. 2b, cells are interfaced with a far-field diffracting wavefront. In order to characterize the intensity of ultrasound that is being delivered to the cells, the displacement profile of the wavefront was obtained with a Polytec UHF interferometer that can measure up to 2.4 GHz range. The displacement profile was obtained with an air interface on the silicon side opposite the transducer. The diffraction pattern (Fig. 1) contains the main lobe and secondary diffraction lobes which are at much lower amplitude, and presumably lower than the intensity needed to cause neural stimulation. The effect of the secondary lobes on excitation will need further investigation, and is potentially another control to adjust the spatial distribution of stimulating forces.

**Figure 4:**
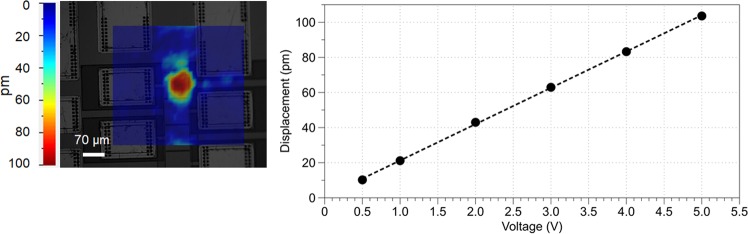
Transducer characterization. (**a**) Displacement profile obtained through the Polytec UHF interferometer in air. (**b**) Peak-to-peak displacement versus drive voltage shows linearity of the piezoelectric AlN transducer. Ten repeat acquisitions averaged by Polytec Software, variance not reported.

The setup for the interferometer data acquisition provided in Fig. 1 consisted of averaging of at least 10 complex valued data acquisitions with a reference channel to provide phase consistency. Data was collected from continuous wave excitation of a 70 *µ*m transducer and displacement magnitude data which is visually depicted in the image. Figure 4 provides a quantification of displacement distribution from the farfield diffracting transducer and the linear voltage response, as expected by the piezoelectric effect. Figure 4a consists of the peak displacement, scanned at a spatial resolution of 10.8 *µm*. The diffraction pattern visual in Figs. 1 and 4a is spatially interpolated for display. Figure 4b indicates the linearity of the AlN piezoelectric ultrasonic transducer for the voltage range used in this paper and provides the displacements that allow for the intensity calculations made in this paper. The continuous-wave frequency response of the transducer wass obtained over at the center of the transducer and used to determine the drive frequency of 1.47 GHz. The frequency response data consisted of multiple resonance peaks, a finite bandwidth, and potential in silicon resonances due to continuous wave stimulation. A drive frequency of 1.47 GHz was used as it is in the approximate central region of the peak frequency response band for the 1–2 GHz drive frequency range. The frequency response was verified by frequency sweep data acquisition using the Polytec UHF interferometer and pulse echo return amplitude. Continuous wave in silicon resonances and multi-layer chip layer stack cause a heterogeneous frequency response. The remainder of this paper contains data for the transducer that is driven at 1.47 GHz. Transducer operation was confirmed with pulse echo measurements prior to use of the device in experiments, with small <50 ns GHz ultrasonic pulses transmitted from the transducer and the reflected signal was measured with the same transducer^36,37^. This is a useful technique in the optimization of finding the exact resonance frequency due to chip to chip fabrication inconsistencies.

### Considerations for the ultrasonic intensity and dissipated energy

The drive amplitude, frequency, modulation, and the duration of actuation determine the intensity and the total energy deposited into the biological sample. It is known from literature that >3 W/cm^2^ intensities induce apoptosis and cellular death *in vitro*^20,29,30^. A stimulation intensity below this threshold by at least an order of magnitude is desired to deconflict the therapeutic window and toxicity threshold. Furthermore, the temperature change due to delivered energy should also be kept small enough such that the non-thermal effects of the ultrasound can be identified.

#### Estimate of intensity

When the incident ultrasonic wave is coupled to a liquid on the surface, a fraction of the incident wave is transmitted into the liquid and dissipates dues to the absorption. The transmitted pressure wave amplitude can be written as ${p}_{t}=\frac{2{Z}_{liq}}{{Z}_{liq}+{Z}_{si}}{p}_{i}$ assuming normal incidence to the surface. *Z*_*liq*_ is assumed to be that of water given the compositional similarity of the media. The transmission coefficient ratio $\frac{{p}_{t}}{{p}_{i}}$ into the cell culture media is 0.135. The pressure in the cell culture media can be modeled as $p(z)={p}_{t}{e}^{-}\frac{\alpha }{{2}^{z}}$. The corresponding intensity in water is $I=\langle pv\rangle =\frac{1}{2}\frac{{p}^{2}}{\rho c}=\frac{1}{2}\frac{t{p}^{2}}{\rho c}{e}^{-\alpha z}$. The absorption of ultrasound in water can be approximated by $I(x)={I}_{0}{e}^{-\alpha x}$ where $\alpha ={\alpha }_{0}{f}^{2}$ and ${\alpha }_{0}=0.134\,\underline{\mathop{\mu m\,ttH{z}^{2}}\limits^{dB}}$ at 40 °C. The transmitted pressure magnitude can be expressed as *|p*_*t*_*|* = *ρcωu*_0_ where *ω* is the radial frequency, and *u*_0_ is the transmitted displacement. The transmitted displacement in liquids will lower than the actual displacement at the surface of the silicon-liquid interface as compared to the displacement measured by the interferometer in air. A peak displacement of ~100 pm was measured in air, as described in the preceding section, corresponding to an intensity of can be used to estimate the maximum intensity in at the water-silicon interface to be 0.3 W/cm^2^. With water loading the actual pressure amplitude in the liquid will be reduced owing to reduced standing wave resonance quality factor and different resonance frequency due to loading. The intensity at the neuron level will be further reduced due the absorption in the media across the silicon-media interface to the neurons^38,39^. In the future, a secondary AlN transducer located in the region of the neuron location can be used to accurately measure the ultrasonic field at the neuron layer.

### Continuous wave stimulation rationale

While pulsed or modulated drive of ultrasonic transducers is often used in inducing neuro-modulatory effects, this paper demonstrates a study of single administration 20 s continuous wave ultrasonics to provide a clear indicator of the effect of this high frequency GHz stimulus without the secondary effect of the pulse train frequency during the excitation window. Given the novelty of the new frequency regime for probing, the effects of neural stimulation in these results are isolated to the GHz frequency regime, without need to decouple effects of pulsed wavepackets that will not represent a single frequency content for the duration of administration. Additionally, using this method there are valid comparisons to other studies using this same cell line^32^. One 20 s stimulus was delivered to the cells during recordings, a longer than usual stimulus time than several other studies to increase delivered energy and match the time scale of calcium ionic transients in order to capture more transients during stimulus. The optical recordings were obtained as follows, minimum of 10 seconds data with no ultrasonic stimulus, 20 seconds of ultrasonic stimulus, minimum of 20 seconds data following ultrasonic stimulus, with a total time under 90 seconds to minimize effects from photobleaching. This duration was chosen to match the time of ionic transients in SH-SY5Y cells reported in the literature^31,32,40,41^.

### Image processing, ionic transient quantification, and statistical analysis

In order to quantify the effect of the ultrasonic exposure on the neural activity, calcium imaging was utilized. Calcium imaging is a well-known method for measuring ionic transients for the chosen cell type and many other neural cell lines and tissue cultures. Calcium imaging was chosen because it has increased immunity to noise due to both cellular and fluid streaming motion artifacts compared to electrode based electrical recording^42^. As described, standard loading protocols for Fluo-8 AM were utilized^43^. Imaging was performed with a digital gain of 900 and frame rate of 45 frames per second using a 340M-USB Thorlabs camera, with slight optimization per sample.

Data collection was performed as follows-10s with no stimulus, 20 s continuous wave ultrasonic stimulus, followed by 50 s of no stimulus. A limited time of data collection was performed to minimize deviations from photobleaching and other similar errors. Experiments were recorded with one actuated transducer directly in field of view at approximately the center of the image and greater than 5 cells in proximity to each other in order to collect data on consistently confluent regions of cells. The glass coverslip itself was at 50–70% cell confluency before starting the experiment.

**Figure 5:**
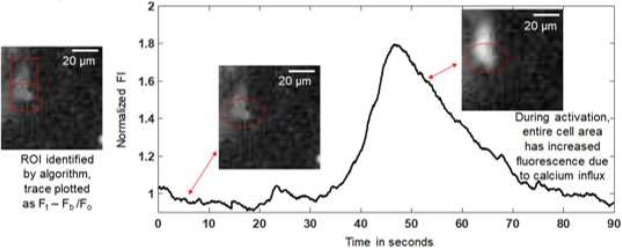
Image processing algorithm. Example trace for one neuron demonstrating calcium transients at 0.3 W/cm^2^ stimulus from image processing algorithm with filtering and smoothing post- processing included. Fb is a region chosen from the data with mostly background signal (not cells). The evolves with the time data, but is constant in region (100 × 100 *µ*m in size). Ft is the fluorescence signal at any time t. Fo is the average value for the first 5 seconds of data collection for each region. Fb is removed at every time point, and the first 5 seconds are averaged to provide Fo.

Cells were identified via thresholding. Figure 5 provides a depiction of one thresholded, identified cell and the calcium transient time course activity, with images of the cell at different time points to illustrate how the algorithm captures stimulation using fluorescence intensity input. In order to analyze the data, normalization was performed by subtracting a background region of 50 × 50 pixels (F_*b*_) to correct for photobleaching and other mechanical, optical, or heat induced effects on the signal that may bias the data. This region was chosen as a region with few to no cells, and was ideally a region with a very small value of relevant signal. All fluorescence intensity levels are scaled to the pixel by pixel average of the initial 5 seconds of data. F_*i*_ are the initial time points of data. The value F_*o*_, which the data is scaled to, is defined as follows, ${F}_{0}\mathop{=}\limits^{\Sigma }{}_{t=0-5s}({F}_{i}-{F}_{b})$. In order to identify regions of interest (ROIs), the beginning and end of the data set was used as a threshold to produce a logic mask that identified regions of cells with consistency (Fig. 5). The normalized change in fluorescence is shown and plotted in local area averages of 20 × 20 *µm* in Fig. 5. The depicted fluorescence intensity was computed as follows $\frac{{F}_{t}-{F}_{b}}{{F}_{0}}$, where F_*t*_ is the fluorescence intensity at any time point t. This quantity is referred to in this paper as normalized fluorescence intensity (FI) and it is unitless. This is the quantity represented in plots and tables for all the data analysis contained in this paper. A 0.5 s moving averaging window was used in order to reduce noise in the time course data depiction and in the time point data analysis. Trials were classified as activated by ultrasound if these two criteria are met−1) an alpha 0.01 significance in difference between the before and after stimulation period (full results in Figure S1) and 2) calcium transients with morphology and fluorescence change indicative of calcium ion flux are present. Further information on the threshold to identify ROIs and classification of activated cells is available in the supplementary methods section.

### Acoustic streaming mechanism evaluation

Acoustic streaming in the fluid alone was modeled with COMSOL. The model utilized the thermo-viscous acoustics module in frequency domain with a user defined frequency at 1.47 GHz and the laminar flow module, with all relevant boundaries set to either slip or no slip depending on the physical location of the boundary in the actual experimental setup. The input of the attenuation dependent acoustic radiation pressure was defined as a volume force, and the driving factor for streaming parameters chosen at each intensity respectively. This setup is supported by previous models^44,45^. The theoretical equations defined by the attenuation coefficient and the far-field Fraunhofer diffraction were inputted into the volume force configuration of the laminar flow module. The below equation for pressure was utilized for this simulation

$$p(x,y,z)=\frac{jw\rho v}{2\pi R}{e}^{-2\alpha z}e-jk[t+\frac{(x{c}^{2}+y{c}^{2})}{2t}]F(rect(\frac{x}{W}))F(rect(\frac{y}{H}))$$

Where p is the pressure, *ρ* is the density of the medium, vis the speed of sound, *α* is the attenuation coefficient, k is the wavenumber, x_*c*_, y_*c*_ is the center of the transducer in the plane of analysis, (x, y, z) is the positional coordinate, W and H are the transducer width and height dimensions, R is the radial position from the thickness, t of the silicon to the coordinate points of interest, F is the Fourier transform, and rect is the rectangular function. The steady-state streaming fields were solved and outputted for the continuous wave ultrasonic stimulus at each power density tested.

Streaming within the sample itself is gauged through the movement of sheared, detached cells under the influence of 20 s of ultrasonic stimulus ranging from 0.013–0.3 *W/cm*^2^. At least 15 velocity recordings were taken per sample from cells ranging in radius from 10–30 *µ*m, with density similar to water. Video data was sampled and positions were found with ImageJ software and displacements were calculated in the dataset for the time points during the ultrasound exposure.

## Results and Analysis

### Differentiation state analysis

Figure 3 provides F-actin staining images to show the differentiation status of SH-SY5Y cells in response to retinoic acid treatment. Figures 3b–d have different morphology due neurite extensions. Cellular branching and extensions are known to be guided by F-actin, and thus the staining and fiber alignment of F-actin is indicative of neurite processes that confer more neural phenotype. These processes and synapse like structures are readily seen in the 48 hour differentiation time-point, along with high cell density, around 50–70% confluency^46^. Figure 3e shows Fluo-8 AM loading of SH-SY5Y neuroblastoma cells indicative of successful loading of cells with calcium chelator agent. Figure 3f,g shows cells immediately before and following ultrasound exposure, with no significant positional and morphological changes with respect to the glass slide.

### GHz ultrasound modulates human neural cells

**Figure 6:**
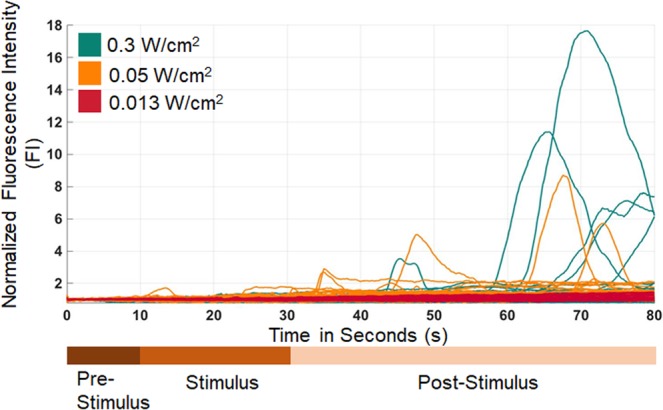
Dosage response and time course data depiction of neurons responding to ultrasound. Intensity dependent calcium transient activity, 0–10 s is baseline no ultrasound stimulus data, 10–30 s is ultrasound stimulus at noted intensity, and 30–80 s is the post stimulus recording period. Time course data depiction for one of each intensity of three repeated trials per intensity.

One feature of this study is that there is an intensity dependence on ion channel stimulatory activity, with insignificant activity levels for ≤0.013 W/cm^2^ ultrasonic intensity and notable activation for ≥0.05 W/cm^2^. At 0.013 W/cm^2^ neural stimulation is statistically insignificant, as can be seen in Fig. 6 and Table 1. Above this intensity point, there is a statistically significant change in the neural activation. In order to discern the influence of ultrasonic exposure beyond the cutoff intensity, mechanistic models need to be constructed. As such, it is difficult to provide an exact curve of the behavior of neural activity given the intensity of stimulus other than concluding that there is presence of stimulatory activity at or above 0.05 W/cm^2^ intensity. Compared to literature values of normalized fluorescence intensity, the values obtained in this study lie in a similar range^32,47,48^.

**Table 1:** Data Summary for Activation and Controls.

Intensity and Condition $\frac{{W}}{{c}{{m}}^{2}}$	Normalized FI for each trial (listed)	Number of ROIs for each trial (listed)	Active ROIs (at t = 70 s)	Alpha Level for Significance Testing	ANOVA tested difference within group of repeated trials	ANOVA tested difference between intensity and condition groups
0.3	1.77 ± 0.616 1.89 ± 0.53 1.933 ± 1.89	130 161 36	23 13 15	0.01	Not Significant	$0.013\frac{W}{(c{m}^{2})}$ and Gentamicin
0.05	1.80 ${\rm{\pm }}\,$ 0.512 1.73 ± 67 2.25 ± 0.9	161 150 57	12 13 29	0.01	Not Significant	$0.013\frac{W}{(c{m}^{2})}$ and Gentamicin
0.013	1.13 ${\rm{\pm }}\,$ 0.2023 1.05 ± 0.54 1.07 ± 0.08	89 25 41	N/A	0.01	Not Significant	$0.3,0.05\frac{W}{(c{m}^{2})}$ and Electrode Free Surface
Electrode Free Surface (0.3)	1.82 ${\rm{\pm }}\,$ 0.13 2.28 ± 1.67 1.77 ± 1.14	25 33 36	10 11 12	0.01	Not Significant	$0.013\frac{W}{(c{m}^{2})}$ and Gentamicin
Gentamicin Treatment (0.3)	1.12 ± 0.34 1.01 ${\rm{\pm }}\,$ 0.18 1.08 ± 0.15	74 30 53	N/A	0.01	Not Significant	$0.3,0.05\frac{W}{(c{m}^{2})}$ and Electrode Free Surface

Data is tabulated for each intensity and condition under which cells are tested. Number of ROIs and ANOVA analysis results are summarized.

**Figure 7:**
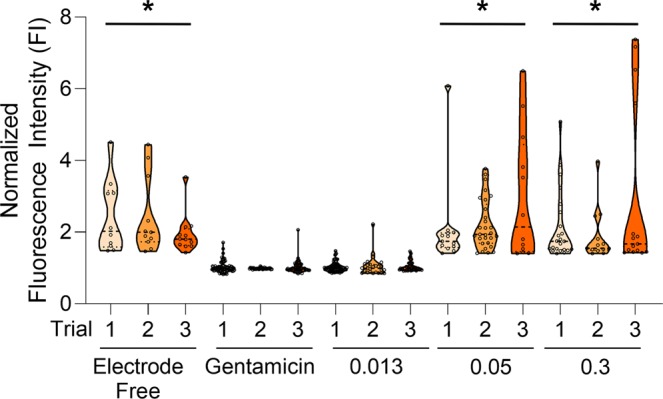
Summary of stimulation results on calcium transients. All samples are depicted as time point average Normalized FI. Number of regions, ANOVA and Tukey Post Hoc provided in Table 1 (error bars in figure = standard deviation). Sample groups with the * have statistically significant activation upon testing with p < 0.01 (tested at a = 0.01). Number of ROIs depicted in Table 1. Electrode Free represents activity with one-sided transducers as control with $0.3\frac{W}{c{m}^{2}}$. Gentamicin data corresponds to sample exposed to ion-channel blocking at $0.3\frac{W}{c{m}^{2}}$. $0.013,0.05,0.3\frac{W}{c{m}^{2}}$ correspond to intensities applied to double sided devices.

**Figure 8:**
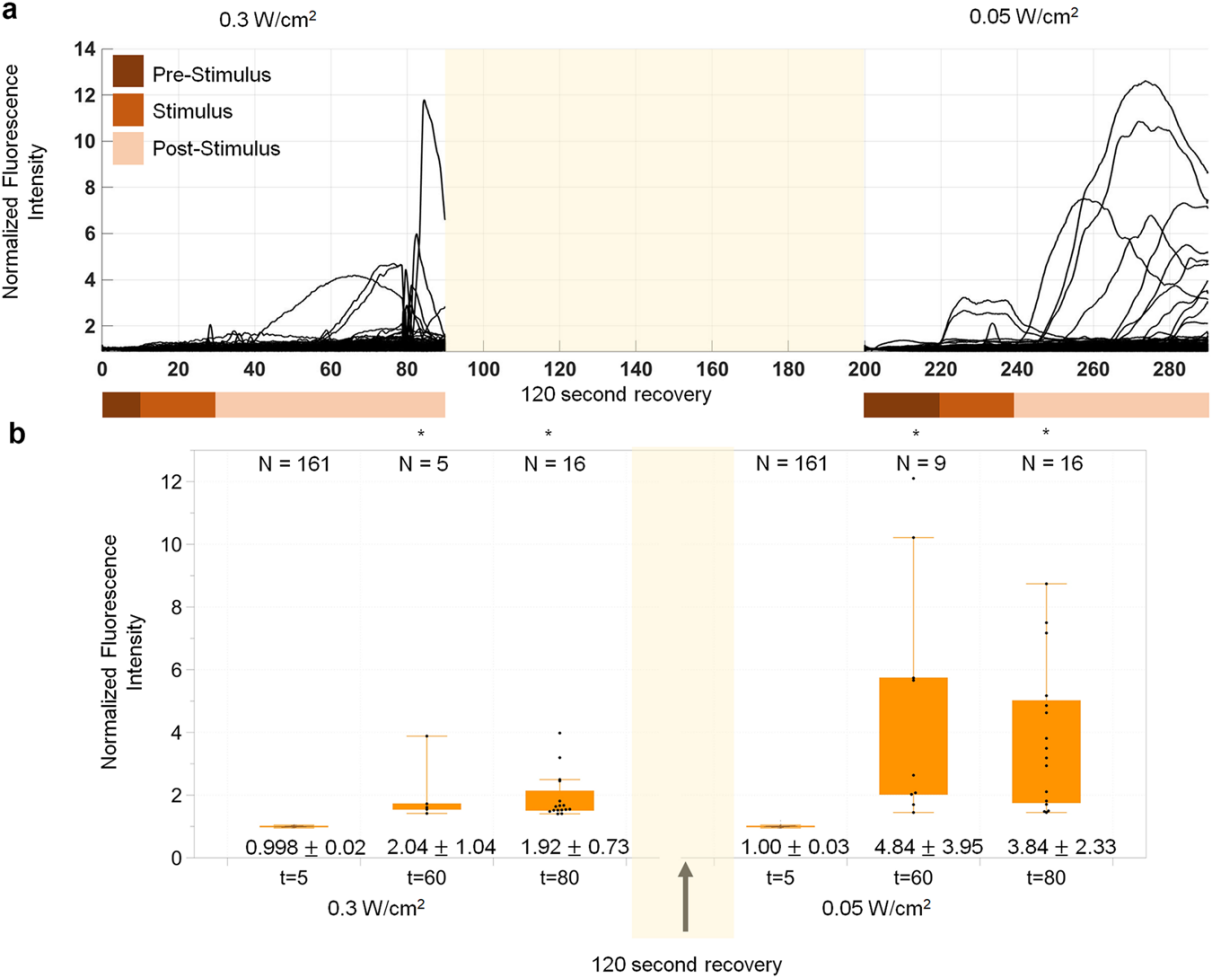
Analysis of cell recovery after stimulation. (**a**) This figure provides activation information of neurons to show the noted activity level recovery with subsequent application of ultrasonic exposure following a 120 second recovery period. (**b**) Number of neurons and mean and standard deviation provided by each box (whisker = IQR). Pre and post stimulus have statistically significant difference upon testing with p < 0.01 (tested at *α* = 0.01). Supplementary Table S2 provides complete ANOVA statistics. Figures with * indicate significant neural activity.

**Figure 9:**
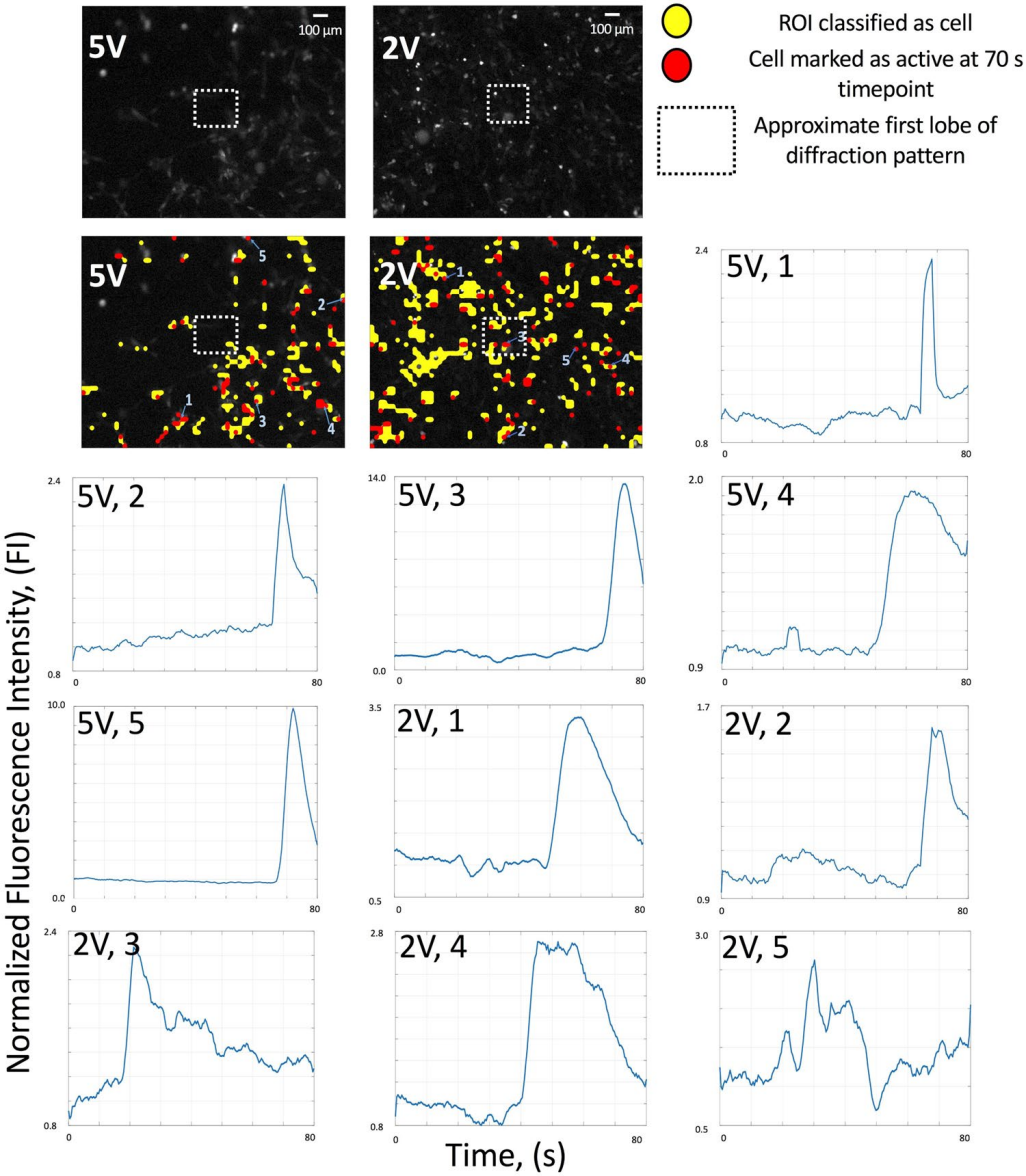
Calcium transient morphology analysis. Calcium transients for individual neurons from Figs. 6, 8, and Table 1 datasets. Only 5 V and 2 V stimulus shown, corresponding to 0.3 W/cm^2^ and 0.05 W/cm^2^ intensities. 5 neurons chosen for each sample that demonstrate calcium ion transients. Neurons in yellow are classified by algorithm as neurons, and neurons in red are labeled as activated at t = 70 s. The corresponding first frame of the multi-frame video data set is shown as a greyscale intensity image for each stimulus intensity. Neurons are labeled as activated either due to a transient at time point t = 70 s. Neurons that are activated and have visible calcium transients chosen (5 per sample) and time course data is graphed to show the calcium transient morphology <0.05 W/cm^2^ data is not shown as these neurons do not demonstrate statistically significant activity and visible calcium transients. These traces are from one of the three repeated trial data sets for each intensity, not necessarily the same trial as depicted in Fig. 6.

This study has two significant effects that can be analyzed – (1) increase in the number and magnitude of calcium transients which is ultimately associated with neural action potentials^40–43,48^ and (2) increase in intracellular calcium levels not necessarily triggering a calcium transient. These two factors are distinct. The first describes a transient event, which is triggered by the activation of voltage gated channels and indicates an action potential. The second many be triggered by many events that are subthreshold, including ionic flux through mechanosensitive channels, membrane strain causing ionic flux, cell to cell channel interactions, to name a few. These are both important as they indicate activity related to the calcium transient due to voltage gated channels or activity leading up to a calcium transient prior to reaching a threshold voltage. Ultimately both of these effects provide information on the ion channel stimulation that is the target of the GHz ultrasonic stimulus. A calcium transient is an indicator of an action potential with associated sodium and potassium fluxes through voltage gated channels^40–43,47,48^. Effect 1 is analyzed in Figs. 6–9, where the activated cell population is compared to baseline levels prior to stimulation and calcium transient morphology is provided. Effect 2 is analyzed in the full population calcium ion flux comparison for the before and after stimulation time points. This analysis for the complete population of cells at two different time points per sample (t = 5 before stimulus, and t = 70 after stimulus) is depicted in Supplemental Fig. S1. Thus, the results utilize and analyze both effects 1 and 2 in the understanding of GHz stimulation of ion channels.

The time course maps provided, as in Figs. 6 and 9, show the calcium transient time course trajectory. It can be seen that while both 0.05 W/cm^2^ and 0.3 W/cm^2^ have similar activation levels when taking values at time t = 70 s, it qualitatively seems that the 0.3 W/cm^2^ case actually elicits a population of calcium ion transients in the time course data that are shorter in duration. This indicates that the associated ionic fluxes that would govern the underlying action potential due to this calcium flux are shorter in duration as well. Following from the ANOVA and Tukey HSD post hoc test on the magnitude of the calcium transients, there are no statistically significant differences between the 0.05 W/cm^2^ and 0.3 W/cm^2^ cases given the analysis criteria, shown in Table 1 and detailed in Table S2 with p-values from the ANOVA analysis listed. In order to determine quantitative differences in action potential duration, further research will need to be performed. The ROI size selection is fixed in size strictly to prevent any data biasing and cell movement effects from falsely inflating the effect of the data analysis, as described in the methods section. The gentamicin calcium channel blocker acts in a similar way as a ratiometric control by negating the presence of other calcium flux (chelator or calcium leaking due to membrane poration or intracellular calcium events) and motion artifacts causing action potential like features in the dataset. In future, research will integrate electrical action potential recordings that will allow for an understanding of the effects of GHz ultrasonics on the duration, peak, and frequency of action potentials rather than calcium transients alone. Action potential duration will be further investigated in future studies on both these datasets and by using patch clamp waveguides to administer GHz ultrasonics and record current from ion channels. This could also provide for more quantitative dosimetry and analysis of time scale events. Figure 6 provides ion flux information, which indicates that GHz ultrasounds stimulates ion channels, for the intensities administered. Figure 9 shows a few of these calcium ion fluxes in detail in order to look at the timescale and duration of these fluxes. Figure 7 displays a comparison of GHz ultrasound activation of ion channels by showing the distribution of normalized fluorescence intensity values before and after stimulus. GHz stimulation ≥0.05 *W/cm*^2^ has significant activation levels after stimulus based off of normalized FI compared to before stimulus.

It is important to note that not all cells are responsive to ultrasonic stimuli, and this is frequently seen and cited with *in vitro* ultrasonic stimulation, especially for this particular cell type^32^. Given that these are cells forming networks of neurons in a intermediate differentiation state rather than being further differentiated with neurotrophic factors, it is expected that there will be a few responsive cells and many that are not responsive to ultrasonic stimulus. Many previous studies support RA differentiation as utilized in this study, but also suggest further differentiation techniques^31,49–51^. In addition, the duration of the transient may be greatly influenced by the modality of stimulation itself. Ultrasonic stimulation may in fact cause slower activation than electrical stimulation. The potential to use this type of system to engineer neural connectivity provides this study multiple interesting, pathways to control *in vitro* neural connectivity.

As shown in Table 1, ratio of total to activated cells may be calculated. It is also notable that a significant number of cells (a cumulative average of 17% and upwards of 40% in some cases) see some level of stimulation. It is difficult to gather a trend from this data other than activation above a certain intensity is present. Given the different force profiles acoustic radiation force, streaming, and other factors play on the stimulation of neurons, understanding stimulation ratio is an area in need of a mechanism centric approach. Table S2 provides relevant complete statistics from the ANOVA analysis at *α* = 0.01 for the data provided in Table 1 of significant stimulation (higher normalized FI) for the different stimulation conditions. The data is analyzed with the log-normal distribution correction described in the methods section for the activated cell population.

In order to establish a reliable control, especially given some expected heterogeneity in differentiation state of the population of cells, the same population of cells should be stimulated with different intensities for an across intensity analysis. However, multiple exposures to ultrasonic could culminate to change the cellular processes, not allowing for a reliable repeat experiment. Thus, recordings from different samples of cells were conducted, following the same culture and differentiation protocol in addition to a repeated trial. The use of independent cell populations leads to some inherent variability in the reactivity of different samples of cells and the repeated trials bolster the analysis by providing similar results to support the conclusion from the previous study. It is plausible that multiple repeat trials establish a trend that can later be optimized for the system of interest. For example, an array of ultrasonic transducers can be used in a tunable manner to activate a network of cells, with a machine learning algorithm implemented to observe the corresponding stimulus to activation relation.

Interestingly, similar studies cite no observable ultrasonic neuromodulation for much higher intensities delivered to SH-SY5Y cells at lower frequencies^32^. Here it is found that cells can be stimulated with sub-apoptosis inducing intensities at the GHz frequency. There are many plausible mechanisms for modulation of neural cells with ultrasound, including mechanical effects such as strain modulation, acoustic radiation force, acoustic streaming force, membrane poration, gene-level and intracellular effects, or thermal effects. At GHz frequencies, due to high acoustic field gradients, these effects maybe more pronounced than at lower ultrasonic frequencies.

In the data obtained it is shown that the mechanisms are most likely mechanical effects rather than temperature or gene level effects. The time course is not suggestive of gene activity modification given the time scale of the neuromodulatory activity. Here the time course of the experimental data is used to rule out blips or puffs, which are smaller time frame (100 milliseconds or less) of IP_3_R mediated intracellular calcium transients^52^. The transients that were observed are on the order of >5 seconds. Differentiated neurons from *in vivo* specimens tend to display calcium transients on the order of 1–2 seconds^53,54^. SH-SY5Y neuroblastoma cells tend to have action potentials that are longer in duration. This is a result of the ion channel densities in SHSY5Y neurons and differentiation state. It is found in this study and other supporting studies that this cell line has calcium transients lasting greater than 5 seconds^32,40^. There will be notable variability of calcium flux morphology amongst cells types and differentiation state, to be taken into consideration and open for investigation in applications of GHz ultrasonic stimulus for other systems^53,54^.

### Ion transient morphology and activation time course in response to GHz stimulation

Qualitatively, a subset of calcium transients seem shorter in time course for the higher in-tensity stimulus. Figure 9 provides visual confirmation of this observation. This could be a result of either greater strain modulation on voltage gated channels during the action potential itself or many neurons reaching threshold voltages more quickly at once, causing amplification of activity during the action potential. The development of patch clamp coupled transducers and electrode array interfaces with ultrasonic transducers will allow for the particular action potential characterization as the control of ultrasonic dosage per ion channel will be better established in this type of system. Ultrasonic stimulation will cause different rates of ion channel activation than electrical stimulation. This is simply due to the nature of the stimulus – voltage gated ion channels may be stimulated mechanically, but the gating mechanism is directly responsive to voltage differential. Similar arguments may be made for other varieties of ion channels. A thorough characterization of this will be detailed in future work.

It is also notable that activity levels are higher immediately following stimulus for the entire recording period, as can be seen in Fig. 6. This is due to the longer time course of a single calcium transient in these cells, the interconnected network of neurons, and the physics of the stimulatory system in potentially inducing threshold towards action potential during stimulus and subsequently causing associated calcium transients to arise post-stimulus. This provides validity to the statistical comparison at 40 s post stimulus at the 70 s time point mark – simply because the cells are more consistently active at this time point across samples. The next section on neural activity recoverability provides some insights on this as a sample is tested under stimulation, then activity is able to decrease post stimulus after a prolonged period of time, then the same sample is tested once again.

### Recovery of neural activity after GHz stimulation

The potential to increase activity of neurons with ultrasonics precedes the question of whether the neurons will return to resting state several seconds after the first stimulus and can also recover activity levels following a second stimulus. To this end, control datasets that show evidence of neural activity decrease two minutes after the pre, during, and post stimulus periods as recorded in Fig. 6, and subsequent reactivation following the same protocol as outlined in the methods section. This data collection consisted of repeated ultrasound exposure as outlined in the methods section with a 120 second recovery period between exposures. This recovery period is not included in the recording simply to minimize photobleaching. The return to resting state of neural activity is characterized in the prestimulus period as per the protocol outlined in the methods section. Figure 8 indicates recovery of neural activity by providing activation levels for a 0.3 *W/cm*^2^ stimulus, then the same activity characterization for a second, subsequent 0.05 *W/cm*^2^ stimulus. There is a 120 second recovery window between the two consecutive stimulations. The time course data provided in Fig. 8 provides before, during and immediately after stimulus activity levels for a 0.3 W/cm^2^ then the same activity characterization for a 0.05 W/cm^2^ stimulus. Before and post stimulation data in bar graphs depicts cells that are activated. The analysis in Fig. 8 shows statistically significant stimulation of cells that were in a resting, non-stimulated state to start with at a 5 V drive (0.3 *W/cm*^2^), then after a 120 second recovery period, cells once again do not show any activation. The resting state is thus recoverable. Again a lower, 2 V (0.05 *W/cm*^2^) drive stimulus is provided. This lower drive voltage is used to show that cells do not develop dosage dependency, and can be stimulated with a lower intensity during the second stimulation. The difference in the ratio of activated cells with subsequent stimulation periods is open for further research. With a 2 V (0.05 *W/cm*^2^) drive stimulus, cells display calcium transients with statistically significant highed normalized FI transient levels compared to pre-stimulus timepoints. The results depicted in Fig. 8 show both potential to recover resting state levels 140 seconds following stimulation and also recover neural activity again with ultrasonic stimulus, providing proof of concept for the use of GHz ultrasonic stimulation for modulation of neural activity. Table S3 provides the results of an ANOVA analysis at *α* = 0.01 for this recoverability test. Time course data is also depicted for an understanding of stimulus distribution across time in both intensity samples.

The ANOVA analysis for the outputs presented in the preceding paragraph and shown in Fig. 8 is provided in Table S3. It is also notable that the 0.3 W/cm^2^ ultrasonic drive showed statistically significant lower stimulation than the 0.05 W/cm^2^. This does not invalidate the claim of recoverability, however it does highlight the potential that within the same population of cells, dosage response studies may be more sensitive, as the analysis for multiple different samples contained higher variability and thus only a threshold type behavior was discovered. It is also possible that the cells are more active due to the first stimulus and quicker to respond to the second stimulus, though the activity does drop down to resting state in between the two samples. This is an area to expand upon in future research, as a larger sample size for recoverability experiments will allow for a more certain and highly sensitive dosage response, important for the clinical and research applications that may employ GHz ultrasonic transducers in future.

### Ion channel blocking confirms GHz mechanism is ion channel mediated

**Figure 10:**
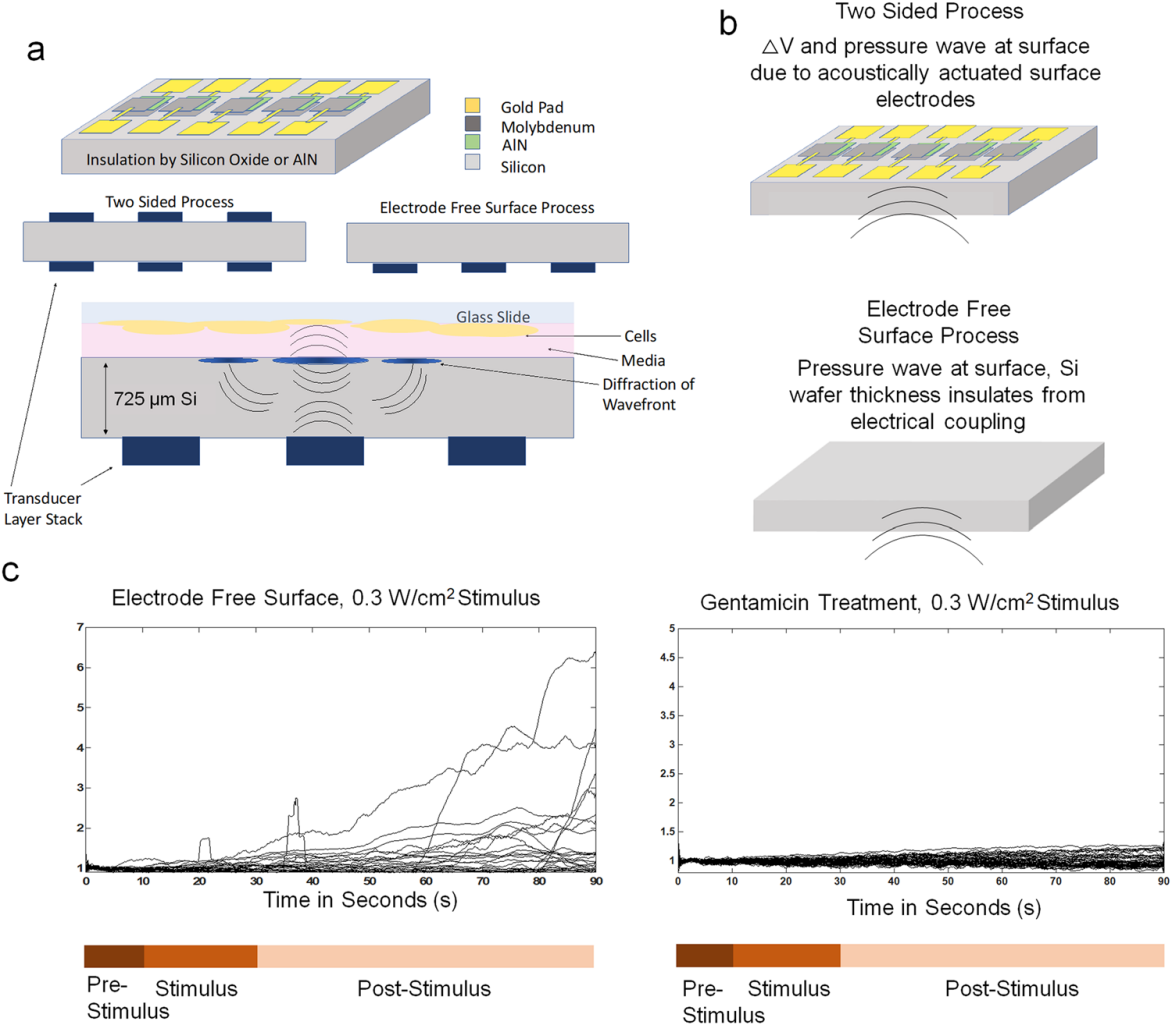
Electrode free surface control (**a**). Comparison of sonically enabled electrodes on surface and electrode free surface setups (**b**). Description schematic of sonically activated electrodes and control used to rule out potential electrical stimulation (**c**). Time course data for Gentamicin and Electrode Free trials depicted to supplement time average data in Fig. 7, with stimulus timings provided.

It is important to negate other plausible causes for changes in fluorescence such as membrane poration, endocytosis, pinocytosis, or other vesicular transport. It is also important to rule out apparent fluxes in fluorescence simply due to the presence of a small scale movement caused by acoustic radiation force or streaming forces. In addition, the potential for ultrasonic stimulation causing time course modification of the normally ~100 ms blips or puffs of IP_3_R mediated intracellular calcium release, thus leading to the potential of conflating these other calcium transient events with action potential activity is necessary to control for^52^. In order to show that the effect of increased intracellular calcium is in fact due to the ionic flux into the cell rather than any other intracellular or transient events, an ion channel blocker was administered. This effectively provides a control that acts as an analog to a ratiometric analysis. Gentamicin (mechanical and Ca^2+^ voltage gated channel blocker) incubated for 1 hour, post Fluo-8 AM loading of cells, is imaged with GHz ultrasonic stimulation and the results are summarized in Fig. 10^34,35,55,56^. Three repeated trials are performed with results summarized in Table 1. There is no time course change in fluorescence indicating lack of calcium flux and absence of induced neural activity. This control establishes that the calcium flux observed is ion channel mediated and not a result of intracellular releases or membrane poration. The notable calcium ion transient morphology noted on a per cell basis for the two significant intensities as in Fig. 9 is indicative that ion channel mechancial stimulation is the salient causation of the activation pattern seen. While there are claims that gentamicin is only a stretch-receptor blocker as noted in studies with the leech^57^, it is noted in other literature that it also effects voltage gated ion channels^58^. It is expected that depolarization occurs through stretch-sensitive channels and voltage gated channels, and perhaps increase the depolarization rate with the activation of voltage gated channels due to increased channel strain. Since gentamicin administration blocks stimulatory activity, this is a good indicator that GHz ultrasonics is inducing ion channel level stimulatory effects, most likely due to the stress waves on the neurons and surrounding fluid.

### Decoupling thermal effects in GHz stimulation studies

**Figure 11:**
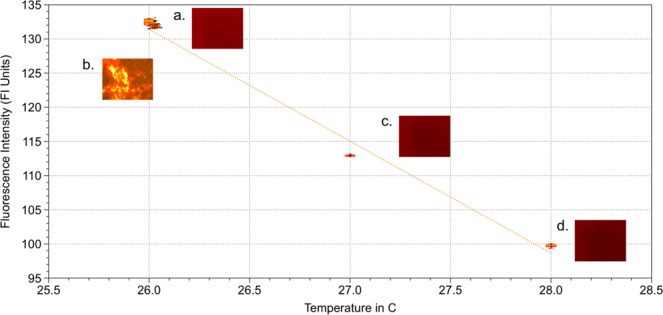
Eu-TTA thermal control. Three temperature points (**a**,**c**,**d**) are taken with imaging agent fluorescence alone for which N = 5 for all 3 groups. The change in fluorescence with ultrasound stimulation for cells is noted, and added to the room temperature settings under which stimulation is performed. (**b**) N = 28 for group. (**b**) Ultrasonic stimulation does not produce significant heating from the 26 C condition. Group b is significantly different from (**c**,**d**), tested at *α* = 0.01, with p < 0.01, while (**b**) is not significantly different from a with p = 0.066.

Potential heating effects of ultrasonic stimulation by the GHz wavefront was quantified with Eu-TTA staining as described in the methods section. The effect of temperature may be estimated using the pressure and attenuation dependent heat from ultrasonic beam and time of stimulus, lending us to minimal theoretical changes in temperature with ranges less than 0.1 °C. The sonic beam induced temperature change can be estimated using $\Delta T=\frac{Q\alpha \Delta t}{{C}_{{p}^{\rho }}}$, where Q is the ultrasonic energy converted to heat produced by the sonic beam, $Q=\frac{\alpha {P}^{2}}{\rho c}$, *C*_*p*_ is the specific heat capacity and *ρ* is the density^19^. This expression assumes no heat conduction from the control volume, and therefore predicts the worst case scenario, maximum possible temperature increase due to acoustic absorption. The theoretical temperature change following this is 0.01 °C. There may be some deviations from the true value within an order of magnitude due to the need for better characterization of attenuation (*α*) and heat induction due to acoustic streaming, intracellular stresses, or other processes. For experimental validation of a minimal thermal effect, the cells are live-stained with Eu-TTA to quantify cellular heating. An ultrasound intensity higher than the stimulation datasets, 1.2 W/cm^2^ at 10 V is administered for 20 seconds to a group of cells loaded with EuTTA. There is a 0.03 °C change in temperature with this administration, (Fig. 11), which is in approximate agreement with the theoretical calculation and it is not statistically significant change from the baseline room temperature. It should be noted that Eu-TTA fluorescence levels change with ACh (Acetylcholine) release also, which should only be exacerbated by ultrasound, and thus yield the most conservative upper limit of thermal effects for this study, enough to support the original hypothesis of mechanical stimulation due to ultrasound rather than thermal effects. Two-sided devices were used in the thermal characterization to provide the upper bound estimate again, because of the potential confounding electrical coupling and communication from the back to front side transducers that would only increase the potential apparent change in temperature. Given the results from this section, a drive voltage of 5 V was chosen as negligible thermal effect on neuron activation was observed. Results are provided with number of ROIs in Fig. 11.

### Decoupling electrical effects in GHz stimulation studies

It is important to rule out any electrical coupling that may be contributing to activity of neurons, especially due to the use of the dual sided transducer devices. It is noted in the literature that high frequency (100 kHz and above) of sinusoidal inputs to stimulating electrode arrays may lead to electrical quiescence of cells^59^. However, this is not a unanimous consensus amongst all sources, and it is important to ensure that the cells are not simply being stimulated with RF GHz sinusoidal electrical inputs. These electrical signals can come from (1) signals coupling from the parasitic electrical fields through silicon and micromachined wire tracks or wirebonds and (2) through silicon ultrasonic communications of bottom side transducers with top side transducers creating electrical fields across electrodes at the top (cell interface) side. In the main devices used, with piezoelectric transducers on both sides of the silicon wafer, the bottom transducers are used to send GHz pressure waves to the top surface and top transducers. This potential coupling effect is illustrated in Fig. 10a,b. The top AlN piezoelectric transducer layer stack can have some associated voltage differential due to the piezoelectric effect, creating a field across the signal and ground electrodes. This would in turn could produce an electric field that could be causing the actuation of the neurons. To control for the effects of stimulation being ultrasonic stimulation rather than electrical stimulation, devices with transducers only on one side were also used. Cell preparation was interfaced with the side that has no transducers, resulting in an insulating 2 *µm* silicon dioxide film and 725 *µm* silicon substrate in between the cells and transducers. Figure 10 indicates that the calcium influx obtained in data as the time point average fluorescence intensity provides statistically indistinguishable activation levels for both one-sided-transducers and two-sided-transducers devices, as shown in Fig. 10c. The calcium transient morphology and number of activated cells, along with the spatial distribution of activation across the sample may very well differ, and this is left for further investigation in future. At present, this paper concludes that ion channel activation is possible with only the GHz acoustic wavefront exciting the cells and associated fluidic streaming. It is possible that the ultrasonically activated electrodes in addition with the administered ultrasound will have unique effects on the cellular activity. At present, this study finds that ion channel stimulation is achieved with both the GHz ultrasonic stimulus alone and the electrical and ultrasonic stimulation together with similar levels of activation. Further research will elucidate the effects of co-stimulation (electrical and ultrasonic) on action potential and calcium flux duration, incidence, and other qualities.

To further eliminate the potential of through-silicon electrical coupling in even the single-sided transducer devices, the bottom side transducers are driven with high voltages (10 V_*peak*_) at off-resonance frequencies (0.9 GHz and 6 MHz). Under these conditions of poor electromechanical coupling, with negligible ultrasonic coupling of waves into the silicon, the calcium imaging data indicates, as expected, that there is no significant neural stimulation. Dual sided devices were tested with off resonance frequency stimulation, and the voltage differential across the top transducers, measured at the contact pads, due to a 5-Vpp RF sinusoidal stimulus at 6 MHz applied to the bottom transducers, was recorded at ≤2 mV peak to peak. This provides evidence that the opposing side of the chip is insulated from electrical activity on one side, thus the electrode free surface devices are certainly free from electrical coupling through the PCB or chip. At resonance, the receiving side transducer has a voltage to voltage conversion for an ultrasonically powered electrode array is approximately linear, with efficiency of energy transmission dependent on frequency response, wavefront diffraction, and device design. This will be detailed in future research more extensively.

### Evaluation of mechanisms underlying ultrasonic stimulus

Many studies with ultrasonic stimulation of neurons employ focused ultrasonic beam stimulation^17–20^, this system delivers far-field diffracting wavefront. The GHz ultrasonic transducer used in this study also delivers a focused beam as a result of a far-field diffraction through the silicon die. Hence, in principle only neuron directly in front of the peak intensity regions of the wavefront should be stimulated. A few mechanisms prevent the conclusive localization of the field to only the exposed neurons. One mechanism is acoustic streaming, in which gradients of the acoustic fields generate non-sinusoidal steady state fluid motion which can generate shear forces on the neurons. Acoustic streaming fluid forces can effect cells distant from the focus of the ultrasonic beam^60^. A second mechanism is the connectivity of neurons due to neural tissue growth, that can lead to excitation from the main exposed neuron to be transported to connected neurons. Given these effects, localization is not manifested in the calcium flux data for the present experiments. Nevertheless, the excitation of many cells has the advantage of providing statistical significance and understanding contributions of acoustic streaming, acoustic radiation force and other factors as separate entities. An interconnected network of neurons is the natural biological tendency of these cells, thus rendering the system to be a better model of neural activity than isolated cells.

One method for pinpointing a definite, frequency and pressure front specific effect is a frequency comparison of ultrasonic effects on the same cell line. Since acoustic streaming and steady state ARF profiles are present with similar order of magnitude values at a different ultrasound frequency, differences in ion channel activity across frequency strongly suggests whether the effects are frequency specific or not. Given the study by Merino *et al*., which stimulates cells from 0.1–0.8 W/cm^2^ at 1 MHz, ultrasonic stimulus alone did not produce any stimulation for this same cell line, suggesting that our studies at 1.47 GHz are uniquely optimal due to the frequency and energy delivery mechanism^32^. We present data that quantifies the effects of streaming in our system, which, coupled with the previous knowledgebase regarding stimulation of these same cells with MHz ultrasound does not result in ion channel stimulation, provides evidence supporting the importance of the frequency specific, direct pressure wave and acoustic radiation pressure itself on the stimulation of ion channels in neural cells.

Numerical simulations in COMSOL result in pressure and velocity of the fluid with stream-lines as shown in Supplemental Fig. S5b,c, with >100 *µm* streaming only near the transducer, with values of 1–50 *µm* streaming or less further from the transducer, particularly >100 *µm* from the center. The simulation does not include results from effects of the cells on streaming. Any difference due to cells is minimal as the acoustic impedance of the cells and surrounding water is minimal. The density of the loose cells is also very low to effect the overall streaming generated flow or the acoustic fields.

The streaming effects plotted in Supplementary Fig. S5a–c show approximate values that would correspond with the fluid streaming velocities derived from the COMSOL model, with some variability given location and heterogeneity of medium and size of cell. The resultant pressure profile is shown in Supplementary Fig. S5c. In future, more intensity levels and configurations will be modeled including turbulence effects near cell layer, in order to better understanding the streaming velocity distribution. In addition, Supplementary Fig. S5a of the numerical COM-SOL simulation shows vortex streaming (white streaming vectors come upwards then return back downwards along the z axis), in a larger geometry shown in Figure S5a to better visualize the streaming vortices. When comparing Supplementary Fig. S5b,c, there are some differences in the model, and higher spatial frequency vortices due to the thin film of water. Simulations on COMSOL of thicker layers of water (200 *µm* thick) show similar larger scale vortex patterns with lower gradient of velocities. In order to better understand these effects, further preparations of cells and interfaces will be compared in the future and matched to the numerical model to better confirm mechanisms. Supplementary Fig. S5d shows at least 15 velocity recordings from each intensity utilized, showing the higher end of velocities observed from the simulation. The data is plotted in conjunction with fluid streaming values from simulation. Since the data is from a detached cell, and the simulation is of fluid streaming alone, the velocities of the two differ as the particle motion is influenced by the particle size, acoustic impedance, and viscous drag, which ultimately influences the model^44,45^. In addition, the simulation is not taking into account turbulent boundary layer effects due to roughness induced by cell layer, and the real streaming profiles are probably much less predictable. The assumption that the acoustic impedance of the interface medium is homogenous and that of water could be under or overestimating the energy delivery of the transducer to the cells, which could be causing deviations, in addition to further nonlinearities from the theoretical equations and due to medium heterogeneity, or due to the unique GHz ultrasound physics. This data will be expanded upon in future, as it is probable that the heterogeneous nature of the culture interface causes deviations from simulation and turbulent flow that induces higher velocities than expected from the simulation. In addition, the modeling of the influence of the time-varying GHz acoustic wavefront will need to be better modeled and simulated, taking into account the GHz ultrasonics interface qualities with further research. This model focuses predominantly on the acoustic radiation pressure dominated streaming profile.

As shown in Figure S5, the maximum streaming velocities from the numerical COMSOL model for 0.0125 *W/cm*^2^ is 60 *µm/s*, 0.05 *W/cm*^2^ is 250 *µm/s*, and 0.3 *W/cm*^2^ is 1400 *µm/s*. While this is certainly an observable and clear physical effect on the cell membrane, it should be noted that most similar papers that employ ultrasound to stimulate neurons and ion channels impart pressures that would induce higher streaming velocities, >1 mm/s^3,4,17,19,32^, and in some cases neurons of the same lineage^32^ are not activated. Thus, it can be inferred that while streaming is a valid mechanism indeed, it is certainly not the only mechanism that is causing the observed calcium transients seen in the data collected in this study. This conclusion is derived from the localization of high fluid streaming velocities from the COMSOL models in Supplementary Fig. S5, and the lower velocities that exist in the majority of the field of view of the cells, supporting data that provides maximum observed streaming velocities, and comparison to other studies in which similar streaming conditions do not induce stimulation of SH-SY5Y cells.

### Data processing control ensures minimal interference from motion artifacts

Using the motion sensitivity methodology outlined in the Supplementary Methods Section 5.3, a representative dataset is shown for two-sided devices at 0.05 W/cm^2^ in S3 (Supplementary Fig. 3). In these control displayed in S3, calcium traces displayed on the motion control graph that exhibit above average intensity values may be exhibiting either motion, influence from nearby cellular motion, or a true ionic flux. As an utmost conservative measure of activation, the subfigure S3c,f removes cells with potential motion involvement from analysis, in which case, activation is still seen in the time course data. Figure S3b,e show the motion control identification of potentially affected cells, both the Gentamicin and 0.05 *W/cm*^2^ show some potential cells for exclusion. These cells are then excluded from Fig. S3a,d and the traces are replotted in S3c and S3f. This analysis provides absolute verification of GHz ultrasound induced ion channel transients by ruling out any potential motion induced artifacts in the data processing, showing that there is still significant activity with the exclusions of any possible movement of cells.

Any cell that produces even minuscule movement is excluded in this analysis, and still there are cells with visible calcium transients, providing a viable worst-case-scenario analysis. This analysis is performed only as a control, as it is too conservative by excluding even potential calcium transients, and the regular analysis outlined in the methods section is used in the rest of the data analysis in this paper as cells are verified against motion artifacts by experimenters prior to data analysis in the standard algorithm outlined in Methods Section 3.9.

## Conclusions and Future Directions

This paper demonstrates one of the first studies confirming GHz ultrasonic stimulation of neural cells *in vitro*. Through the use of CMOS compatible chip-scale ultrasonics, this paper provides a foundation for a possible localized, closed loop, immune compatible neural prosthetic, for use in treating, understanding, and even engineering neural tissue. This paper reports on the direct mechanical effects of GHz ultrasonic stimulation on ion channels, negating possible electrical or thermal effects. In the future, further work is needed to identify the mechanisms of the mechanical stress waves on the neurons. A variety of force profiles are at play aside from the incident pressure field. While the stimulus can be localized to a single neuron, acoustic streaming is induced as an intensity dependent process that can affect further away neurons. These various mechanisms (acoustic streaming, acoustic radiation force, other forms of strain modulation) all impart unique force profiles on the neural cells that are difficult to quantify by using simply intensity as a measure of sonic stimulation^60^. Isolating stress wave exposure to micro- to nano-scale volumes can be achieved by patterning a highly absorptive layer facing the sample. Alternatively, phased array techniques and transducer designs can be devised to further allow for localization through computational optimization. Further research into sample preparation such that neurons are unconnected to each other, or the use of high speed cameras to capture neuron excitation propagation may be needed. Acoustic streaming can be prevented by seeding neurons in restrictive microfluidic chambers and microstructuring onto the chip scale device that can cause fluid damping to prevent high fluid streaming rates. In real brain tissue, the interface will be less impacted by fluids surrounding the cell due to the packing density and matrix nature of brain tissue.

This paper describes experiments in which the sonic intensities are chosen to be under the known limit of apoptosis induction and toxicity thresholds and within known stimulatory ranges, however for applications of cellular ablation and tissue interfaces, a phased array configuration and concentrated transducer designs make it possible for upwards of 100 W/cm^2^ onchip surface intensities to be administered to cells. A thorough characterization of toxicity of GHz ultrasonic stimulation, in addition to the effects of this frequency regime on cellular structure and morphology, can also be investigated for further functional control of neural tissue.

Since GHz transducers can be integrated within CMOS, this technology can lead to fully integrated GHz ultrasonic microsystems that can be used for clinical use and research methods for neural systems. Compared to other neuromodulation approaches, ultrasonic neuromodulation is label-free and can be conducted without the lifetime limiting due to tissue encapsulation of electrode based devices. Compared to other lower frequency ultrasonic approaches, the high frequency GHz operation has the potential of achieving sub-neuron ultrasonic energy confinement. The high frequency generation from the side of a chip opposite to the sample allows one to form specific beam profiles to address different neurons at different times to control temporal and spatial stimulation. Furthermore, by using transducers on the opposite side of the biological sample side, electrically isolates the electrically active side from the biological side.

The development of new technologies for applications in neural prosthetics begs the question of how to target the current challenges. In the introductory section, current challenges in prosthetic development were summarized and the potential for GHz ultrasonic chip scale systems in addressing these challenges was detailed. In addition to the theoretical potential for GHz chip-scale devices as the new contender in the arena of neural prosthetics, the results of this study provide mechanistic information and threshold behavior that suggests further potential. Supplementary Fig. S4 shows a potential implantable, wireless chip-scale GHz ultrasonic neural stimulatory device. Supplementary Table S4 provides a column on current device limitations in vagus nerve stimulation, and the current challenges due to poor localization and variable effects. The vision for GHz stimulation is born from the energy localization, low power requirements, CMOS-compatibility, and low toxicity of GHz ultrasonic stimulus. This is the future vision for the use of GHz ultrasonics in therapeutics. The results from this study indicate that there is a dosage sensitivity of neural cells to ultrasonics in terms of ion channel stimulation. Further information using electrical and thermal controls indicate that the stimulation mechanism is most probably mechanical. Ultrasonics may have many effects on neural cells, from cellular differentiation, matrix remodeling, and regeneration to ion channel stimulation^61–63^. The focus of the results in this study provide venues for ion channel stimulation using GHz ultrasonics. It is possible that, using the knowledge of this threshold type behavior, systems can be designed to delivery a certain dosage under which neurons primary remodel and regenerate, and above which they primarily are stimulated (or perhaps both stimulated and remodel). Further work is being conducted on neural differentiation and re-structuring under GHz ultrasonic exposure. As such, the threshold type behavior is very useful in providing multi-functionality for this chip-scale system.

In order to better model the physical effects of GHz ultrasound exposure on the ion channels, future research will be looking into the use of ion channel blockers and genetically modified cell lines that do not have mechanosensitive channels. Recent studies have cited the effects of ultrasonic stimulation – a combination of both acoustic streaming and acoustic radiation pressure, along with the direct pressure field, as being influential on the mechanosensitive ion channels alone, and having much less influence on purely voltage gated ion channels. Other studies cite that neurons which are a part of excitable tissue are more excitable under ultrasonic exposure due to the simple strain modulation being an additive force. In order to understand the particulars of the physics of the GHz system, knockout cell lines will be established that do not contain the important mechanosensitive channel players (PIEZO1, TRAAK), and additionally transfected cell lines will be utilized that have differing ion channel expressions, and perhaps even differing quantities of expressions of ion channels in the membrane, and stimulus response will be compared. According to the human protein atlas, SH-SY5Y has a low RNA expression of PIEZO1 of FPKM of 1.5–2.1, a relatively high expression of TRAAK (from expression of subunit KCNK4), both mechanosensitive ion channels. Since calcium transients are induced with any potential transmembrane voltage change, it is possible that ultrasonic stimulation has a higher effect on the mechanosensitive ion channels present in SH-SY5Y cells rather than the voltage gated ion channels^64^. There have been numerous studies targeting different subtypes of ion channels. In order to provide mechanism and threshold insights, we will evaluate GHz stimulation further in cell lines with either enhanced expression of certain ion channels or knockout cell lines, either obtained as stable lines, or transiently altered through transfection. Example work by^65^ recently showed the potential for controlled ion channel expression through tetracycline regulation. Recent studies^66^ show the potential for comparative membrane potential changes with and without certain populations of ion channels. Additional research^67^ introduces the HEK-P1 cell line which uses a CRISPR/CAS9 knockout of the PIEZO1 mechanosensitive channel, with no significant currents due to mechanical activation. The same study also utilized an enhanced PIEZO1 expression in HEK293T cells through the use of Piezo1 chimera transfection. Similar protocols will be carried out in future GHz studies in order to understand and isolate stimulation effects to certain ion channel populations. Please refer to Supplementary Table S4 for a review of past and present methods in neural stimulation. Additional relevant literature is summarized in this table^68–92^.

Other future directions include computational modeling and mechanisms of stimulation to be supplemented with experimental data and measurement of action potentials electrically using an electrode array formed on the side interfaced with the neural sample. Further research on ultrasonic effects of cellular restructuring, toxicity, differentiation, and sonically powered electrode arrays is in progress. This study opens the door to many new possibilities for engineering, treating, and understanding neural tissue.

## Supplementary information

Complete Supplementary Material.

2V RF excitation.

5V RF excitation.

Excitation with one-sided transducers.

Excitation with Gentamicin.

## Data Availability

Sample data sets from 4 different trials are provided. Cells that are representative of activity (or lack thereof) in the sample depicted are selected by region and provided. The video data is presented at 8X speed, with slightly longer prestimulus period (~20 s) in order to provide a better visual of resting state. Prestimulus, during stimulus, and post stimulus is labeled during the video. Data is raw and unprocessed, other than selection of a region of interest with a few cells and addition of labels of sitmulus time points. Movie 1–0.3 *W/cm*^2^ depicts data from the 5 V drive with cells depicted that are stimulated by the ultrasonic exposure. Movie 2–0.05 *W/cm*^2^ depicts data from the 2 V drive with cells depicted that are stimulated by the ultrasonic exposure. Movie 3 - Gentamicin depicts data from cells that are not stimulated. Movie 4 - Electrode Free Surface depicts data from cells that are stimulated by GHz ultrasound free from electrical coupling due to surface devices (one sided device controls from the results section). These representative datasets are provided to show stimulatory activity from raw data sets. Further data will be available upon reasonable request.
