# Supplementary material for: GHz Ultrasonic Chip-Scale Device Induces Ion Channel Stimulation in Human Neural Cells
Source: Sci Rep. 2020 Feb 20;10:3075. doi: 10.1038/s41598-020-58133-0 (PMC7033194; doi:10.1038/s41598-020-58133-0)
Supplement: Supplementary file 1 — Complete Supplementary Material. [file 41598_2020_58133_MOESM1_ESM.pdf]

GHz Ultrasonic Chip-Scale Device Induces Ion Channel Stimulation in Human Neural Cells

Priya S. Balasubramanian[1], Ankur Singh[2,3], Chris Xu[4] & Amit Lal[1,*]

[1]*School of Electrical and Computer Engineering, Cornell University, Ithaca, 14853, NY, USA*

[2]*Sibley School of Mechanical and Aerospace Engineering, Cornell University, Ithaca, 14853, NY, USA*

[3]*Meinig School of Biomedical Engineering, Cornell University, Ithaca, 14853, NY, USA*

[4]*School of Applied and Engineering Physics, Cornell University, Ithaca, 14853, NY, USA*

[*] Amit Lal; amit.lal@cornell.edu

Emails: psb79@cornell.edu (Priya S. Balasubramanian), as2833@cornell.edu (Ankur Singh), cx10@ cornell.edu (Chris Xu)

Supplementary Methods & Discussion

Image Processing, Ionic Transient Quantification, and Statistical Analysis

The aforementioned thresholding to identify cells is employed by identifying ROIs with normalized fluorescence intensity above a certain threshold. This threshold is usually selected post background subtraction to be within the range of 0 to 80. The decision of threshold is dependent on the SNR of the sample. For each sample, thresholds within this small range are tested to identify which value best segments each ROI in the image and identifies cells. The thresholds are set and the segmentation is studied and compared visually in order to verify the correct choice of threshold. A second threshold is applied after the analysis is complete to only analyze cells that retain reasonable SNR throughout the recording period. This threshold is chosen to be approximately the same in each sample, restricted to a small range of a normalized FI of 0.85 to 1.0 at a time point during the end of the time course data. The threshold is chosen within a small restricted range simply because potential, small, and unforeseen differences in loading conditions from sample to sample may slightly change the SNR of the system and resultantly the photobleaching effects over time, and the value that captures most cells with sufficient SNR is identified through validation with actual image frames.

The difference between activated cells after ultrasonic stimulus and the population of identified cells prior to ultrasonic stimulus is analyzed as well as distribution similarities between repeated data sets. An activated cell is defined by analyzing the entire population of cells during the post stimulus period. The entire fluorescence intensity average for the population is taken, and a threshold slightly below this average value is chosen ($\mu - 0.2\sigma$). This threshold is found to be

1.4 (FI normalized unitless fluorescence intensity). In some data sets, some outlier cells that skew the distribution away from Gaussian may be removed for a more accurate application of ANOVA analysis. Correction is performed by restricting values to be within a deviation of the mean. It is ideal to prevent inaccurate skewing of activation due to one cell producing an unusual response as the ANOVA analysis is only accurate when applied to normal distributions (section 5.2 expands further on this). Cell preparation samples with improper calcium loading are disregarded due to sequestration and other effects visible in the field of view. If 30% of cells have suboptimal loading, the sample is not included in the analysis. In addition, samples with ample cellular debris that appears and moves in the field of view are also disregarded. Sample with small amounts of cellular debris that move due to fluid vortices that exist at the time of ultrasonic stimulation are checked for interference due to this cellular debris and mobile debris are not segmented as cells. In addition, cells in this sample that may be influenced by moving debris are excluded from analysis. The lack of neural activity-like transients in the control samples, despite the potential debris and microscale cellular movements, is a good proof of concept that this algorithm does not falsely produce action potential like transients which are in fact due to other ultrasound induced effects or movement artifacts.

For the analysis, a time course of activation of ROIs sized 20μmx20μm was devised, and then obtain a single time point and average all ROIs in this time point in the prestimulatory period (from 0-5 s) and similarly choose one time point after the stimulus is applied (70 s) and average all ROIs in this time point. The second time point is chosen as a time point when activity levels are qualitatively high for all samples that show any activity level. Averages are depicted graphically. Statistical analysis and normalization techniques are detailed in the next section. In order to establish a difference between the before and after stimulation periods, a strict 0.01 alpha level was enforced. Though not very visible in the samples analyzed, movements of the cells given ultrasonic exposure is a potential cause for false increase in readings, and this strict alpha requirement was enforced to mitigate the potential of errors derived from the motion. The datasets analyzed in this paper demonstrate negligible movement of cells due to ultrasonic stimulus given good adhesion of cells to surface and low intensity ultrasound application. The ion channel blocker administration provides another control for this movement effect.

Trials are classified as activated by ultrasound if these two criteria are met - 1) an alpha 0.01 significance in difference between the before and after stimulation period (full results are shown

in S1) and 2) calcium transients with morphology and fluorescence change indicative of a calcium transient are present. Sample are thus analyzed not only for the presence of elevated intracellular calcium levels, but also for actual transient activity. Morphology and time course of calcium transients is comparable to ultrasound facilitated exposure induced transients in SH-SY5Y cells in the literature [32] . In each sample classified as activated, traces of cells with transients indicative of ion channel activation can be seen, as shown in **Figure 9**. This prevents misclassification of samples as activated that are simply showing fluorescence increases due to effects of increased calcium localization and sequestration or unrelated, potentially ultrasound induced effects. Activated neurons are defined as those with fluorescence intensity cutoff determined by the population mean fluorescence intensity and standard deviation, with a protocol using similar criteria (mean and standard deviation for determination of activated cells) followed in a similar study of the same cells exposed to different frequency ultrasound and electrical stimulation [32, 43]. Three trials per condition are analyzed. An ANOVA analysis with Tukey HSD post hoc test with 0.01 alpha is conducted to compare activations from each trial, wit normalcy of distribution tested and determined to be normal either as is or with log transformation (log-normal distribution), as detailed in section 5.2.

Statistical Analysis

In order to perform the first order comparison within a sample to classify it as activated or not activated by ultrasonics, a t-test was performed assuming normality of the fluorescence intensity of the complete population of cells. This assumption is justified by both quantitative testing of the samples described in this paper and previous research. In order to assert the normality of the full cell population, a total of 1292 cells are analyzed prior to ultrasonic exposure and the fluorescence intensity is recorded via the image processing methodology outlined in the previous section. The normalized fluorescence intensity values of the cells are plotted in S2a. This distribution is compared to a normal distribution with the null hypothesis of similarity between distributions. It is determined at a $p = 0.4745$ to accept the null hypothesis at 0.01 significance level that the data is normally distributed using the Kolmogorov-Smirnov test for normalcy. Thus is order to compared time points within the sample, t-test and ANOVA statistical analysis may be used without further normalization of the data. This finding is further bolstered by previous research on the subject, which takes into account both the distribution of the electrical recording of action poten-

tials, which translates to ionic flux in optical recording, and the firing rate [68] . This phenomenon is also relevant in the current analysis. Once cells are activated, the activated population alone will certainly demonstrate at the least a skewed normal distribution, if not a multimodal, more complex distribution as cells are not all homogenous in the sense that they are differentiating towards an end phenotype. This is visible as left-skewing in S2c and S2d. The data plotted in S2c and S2d is the entire population of cells at an activated time point (t=70 s). The data, without normalization, fails to match a normal distribution at a count of 119 cells with $p = 0.0004$ to reject the null hypothesis under the Kolmogorov-Smirnov test. Given this, in order to perform an ANOVA amongst all trials and samples, the distribution must be corrected to obtain normalcy in order to employ statistical tools. While the complete characterization of calcium imaging fluorescence value distribution in SH-SY5Y cells during or after stimulation at different differentiation time points is left for future research, using enlightenment from previous research and statistical analysis of datasets, it is determined that the data of activated cells alone, after removal of major outliers, follows a log-normal distribution. In order to normalize this distribution, the log of the data is taken as a comparative measure [68, 69]. It is noted the neural cell populations often have skewed distributions for a variety of reasons in previous literature [68]. Following normalization through log operation on the dataset, the distribution matches a normal distribution with $p = 0.7876$ to accept the null hypothesis under the Kolmogorov-Smirnov normalcy test with 119 cells total as shown in S2b. It should be noted that log normalization of the normally distributed nonactivated cells does not disrupt the normal distribution, allowing for comparative metrics to be employed among samples that are activated and not activated using the same transformation.

Motion Sensitivity Controls for Image Processing

In the case of cell motion, floating debris, and other similar movement related image processing artifacts, the most conservative measure of the potential activation is reported as a control. In order to obtain a conservative measure of calcium flux, that rules out all potential movement related artifacts, all frames of the time course images are thresholded as described in the previous section (the first threshold), then processed as binary. This type of processing methods can capture both calcium transients (given that the threshold may capture different cells in different regions depending on the fluorescence intensity) and motion artifacts, but it is more sensitive to the motion artifacts and will capture all motion artifacts. By running this analysis and verifying that any cells

captured to have transients in the binary representation of the image processing algorithm should be removed from the analysis, a second analysis can illustrate ionic flux events from cells that absolutely are not influenced by any ultrasound induced motion or moving cellular debris. This increases the certainty that the activation is indeed from only what could be increase in fluorescence signal within a cell due to Fluo-8 AM chelator flux rather than other artifacts. The data depicted in Table 1 and the accompanying figures is from the algorithm analysis in section 5.2, given that this motion sensitivity control is a very conservative measure of calcium flux and probably excludes real ion fluxes due to the nature of threshold based processing. As such, the method illustrated in this section is used to only verify that, with full certainty, the samples of interest do display activation that is certainly not a result of motion or motion induced fluorescence signal artifacts.

Applications of GHz Stimulation to Vagus Nerve Stimulation

The comparison presented in **Supplementary Table S4** provides motivation to reach higher resolution metrics with a low toxicity, immune-compatible method. As can be seen in the row on GHz ultrasonic stimulation, most of the previous work has been done for imaging cells using GHz waves and not in the realm of neural cell stimulation and structural characterization. Supplementary **Figure S4** illustrates the potential for this type of system in peripheral nervous tissue, with different localizations of the wavefront shown with the chip-scale device configuration. Here, GHz ultrasonic phased arrays can focus onto specific axons. The GHz sonic chips can be injected and powered using RF power transfer. The localization of the sound field prevents unwanted effects outside of the region of interest (e.g. nerve sheath). For lower frequency modalities of ultrasonic stimulation, nonlocalization outside of the nerve sheath can cause heating of tissue, vascular involvements, and other neural involvements, complicated the potentially straightforward intentions of, for example, vagus nerve stimulation. In **Supplementary Table S4**, vagus nerve stimulation as a clinical need is briefly described, with ultrasound methods at present suffering from variable effects (stimulatory or inhibitory), partially due to lack of localization to a single nerve fiber, while optogenetics and electrode arrays suffer from toxicities and lack of longevity respectively. While kHz-MHz ultrasonics represent clear lack of localization limited by effective wavelength in tissue, electrode arrays suffer similar localization due to wire interface with tissue. Classically, electrodes are placed as on-chip contacts or wire wrapped to interface with tissue, both resulting in fabrication limited localization and penetration. GHz ultrasonics with beam steering and focusing can more

effectively and precisely deliver energy to a target tissue. Optogenetics methods, while highly useful in research settings, require genetic modifications that present toxicities and hamper clinical translation.

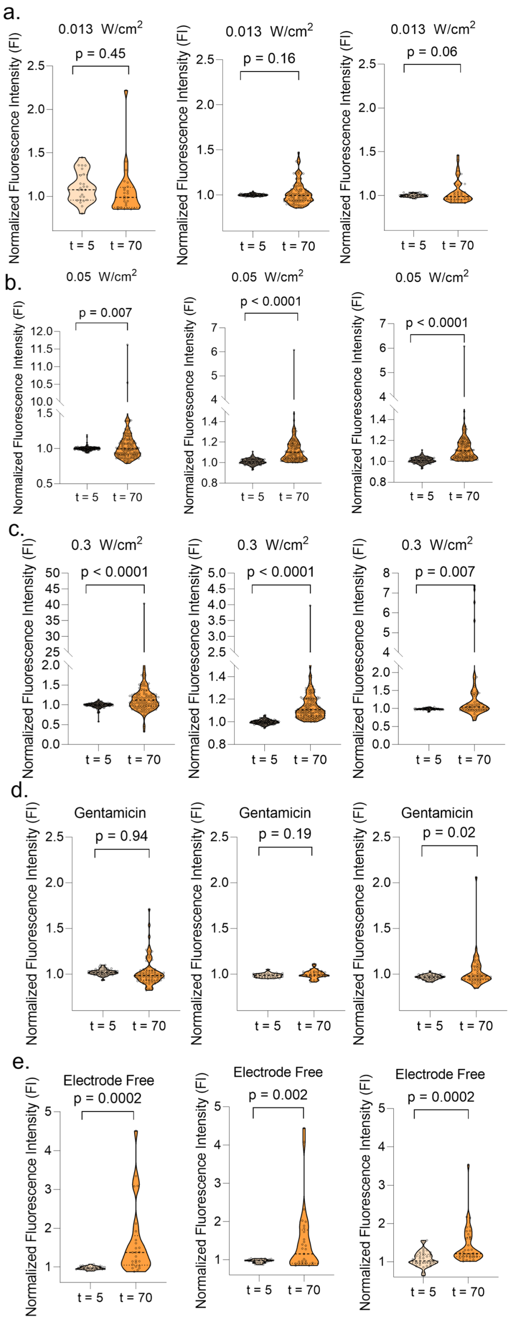

Supplementary Figure S1: Pre and post stimulus activation time point analysis. Activation analysis for all samples analyzed. Entire population of cells analyzed before (t=5) and after (t=70) ultrasonic exposure, with all cells included in this analysis. Activated cells only summarized in **Table 1**, **Figure 7, Table S2**.

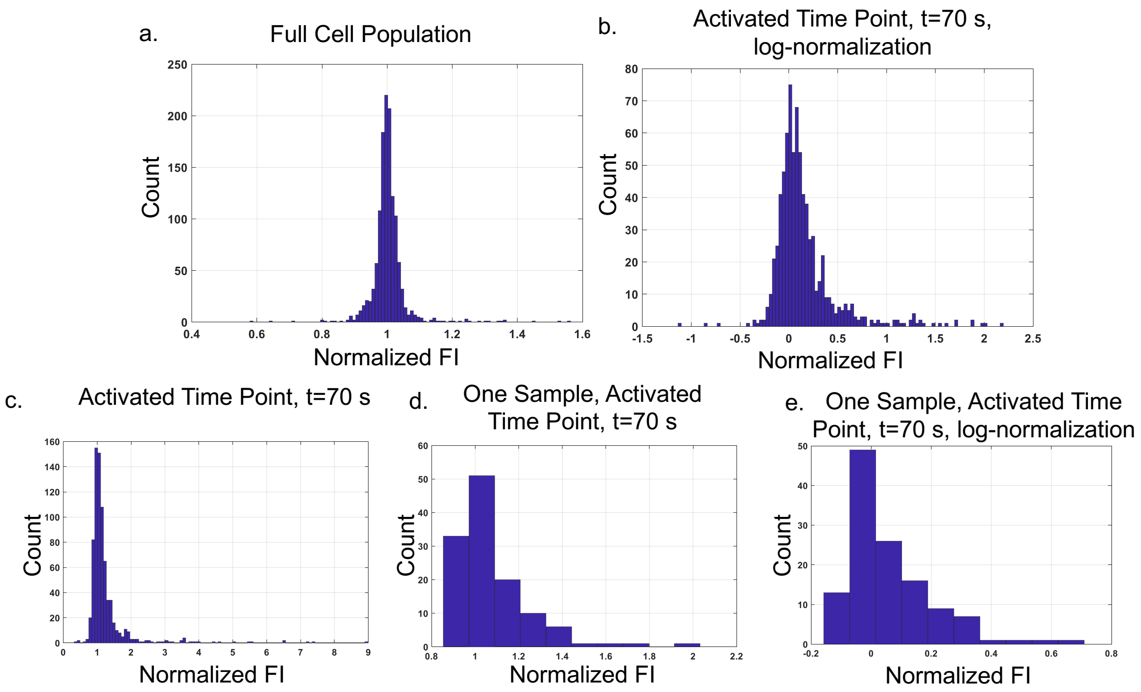

Supplementary Figure S2: Neural cell normalized FI distribution analysis. a. Distribution for all cells analyzed for normalcy with n = 1292. It is determined at a p = 0.4745 to accept the null hypothesis at 0.01 significance level that the data is normally distributed using the Kolmogorov-Smirnov test for normalcy. b. Log-normal distribution fits data of activated cell population during the activated timepoint of t=70 s. The log normalized activated cell only distribution is shown in this subfigure. Following normalization through log operation on the dataset, the distribution matches a normal distribution with p = 0.7876 to accept the null hypothesis under the Kolmogorov-Smirnov normalcy test with 119 ROIs total. c. Distribution of activated cells only fails to match a normal distribution at a count of 119 ROIs with p = 0.0004 to reject the null hypothesis under the Kolmogorov-Smirnov test. The distribution from b is shown without log-normalization. d. Distribution of activated cells (cells at t=70 s) plotted for one sample alone, without log-normalization. e. Analysis in d performed with log-normalization. Left-skewedness is corrected through the log normalization of the data.

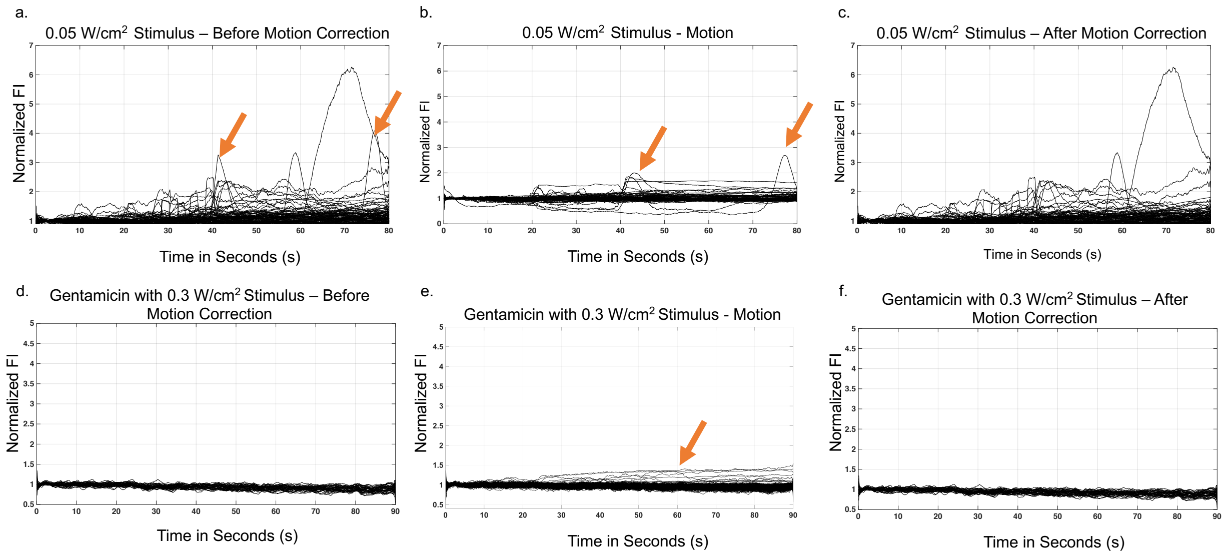

Supplementary Figure S3: Motion Control Analysis. Worst case scenario analysis to exclude cells that might at all be influenced by cellular motion. a. depicts before motion correction, b. depicts which cells may be influenced by motion artifacts, and c. excludes these cells from the final compiled time course dataset for 0.05 W/cm^2. d. depicts before motion correction, e. depicts which cells may be influenced by motion artifacts, and f. excludes these cells from the final compiled time course dataset for Gentamicin controls stimulated at 0.3W/cm^2. Because cells are visually analyzed for motion, and the contribution of motion is very low, this analysis is used as a control only, and is not applied to the datasets analyzed throughout this paper.

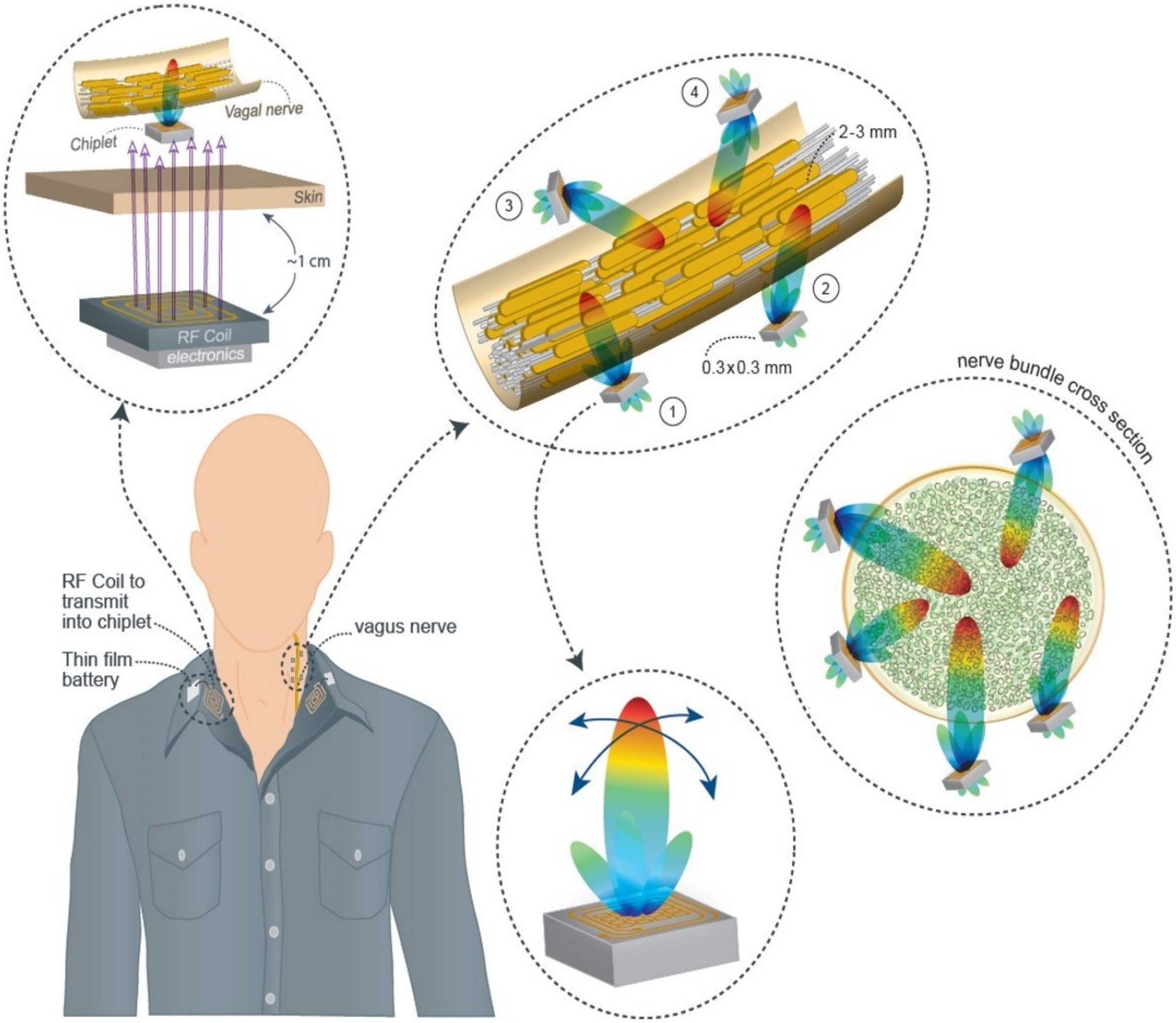

Supplementary Figure S4: Concept for implantable GHz sonic neural prosthetic chips. The potential for multiple CMOS integrated, GHz ultrasonic chips are depicted. This can be used in the central or peripheral nervous system, and other organ systems to achieve axon specific neurostimulation. The interaction of the sonic waves and the resulting diffraction pattern with tissue is depicted. Numbers 1-4 on the top right indicate potential spatial configurations that allow for beam steering and precision delivery of ultrasonic energy.

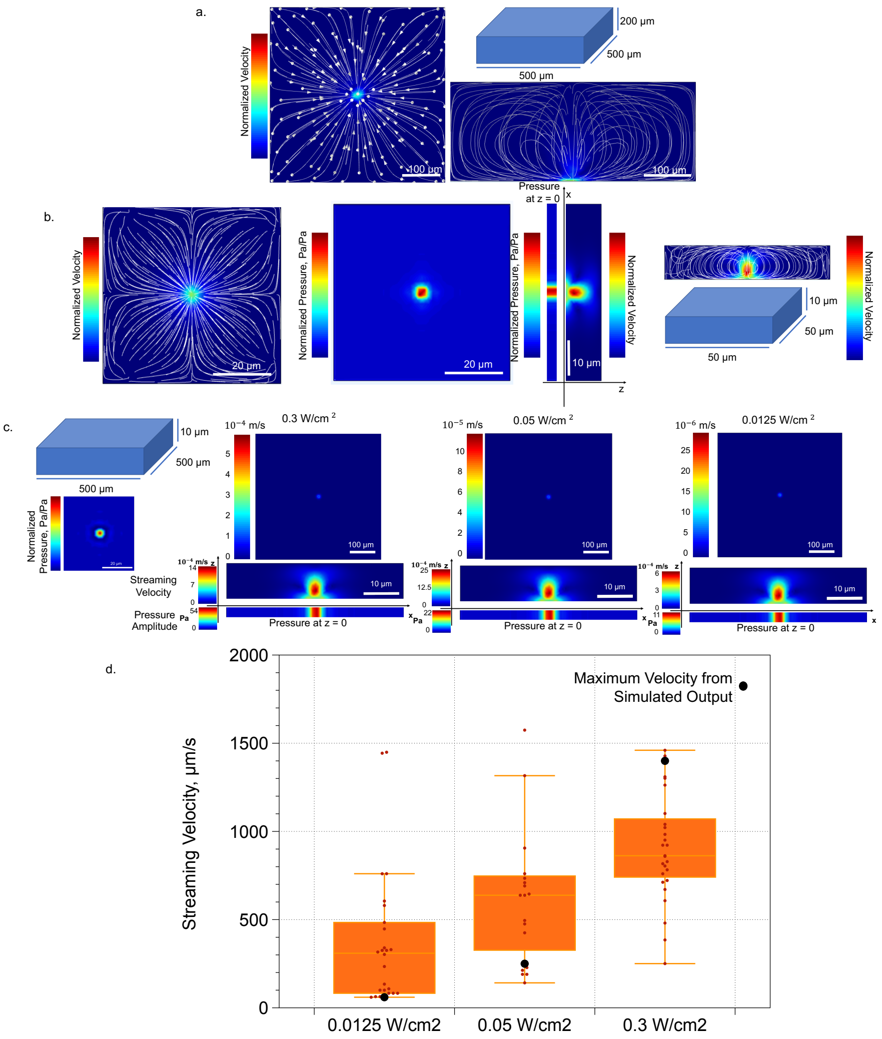

Supplementary Figure S5: Initial modeling of acoustic radiation pressure induced flow.
a. Acoustic streaming due to radiation force in a larger cavity (500 x 500 x 200 μm) simulated in COMSOL using a radiation force calculated from the absorbing field, xy and yz cross-section illustrated with normalized velocities. Larger cavity used to better show streaming lines associated with vorticies. b. Geometry used in simulations and results in figure b through d, same as what is utilized in the experimental sections of the paper, smaller subsection of entire region modeled in b for higher simulation accuracy. Cross section of velocity shown with streamlines, pressure profile shown for xy cross section, then extracted at x = 0, z = 0 and depicted with associated xz streaming velocities plotted for chamber. c. Results from b depicted for the entire imaging window, providing localization of streaming velocities given the transducer geometry, in xy crosssections. d. Experimental results from streaming using cell tracking with high speed camera, at least 15 velocity recordings per data point. Velocities derived from maximum points of the simulations also plotted for reference. The results from the simulation are from fluid streaming velocities alone, whilst data points from streaming of detached cell.

Supplementary Table S2: ANOVA Analysis Amongst all Trials. Unpooled analysis amongst all trials to show significance at $\alpha = 0.01$. Data is log-normalized given analysis presented in methods and results sections. T designates trial, astericks designates significance at given alpha level.

Comparison Groups ($\frac{W}{cm^2}$)	P-value	Significance at $\alpha = 0.01$
0.3 T1 vs One-sided T1	0.98506	
0.3 T1 vs One-sided T2	0.97891	
0.3 T1 vs One-Sided T3	1.00000	
0.3 T1 vs Gentamicin T1	0.00000	*
0.3 T1 vs Gentamicin T2	0.00000	*
0.3 T1 vs Gentamicin T3	0.00000	*
0.3 T1 vs 0.3 T2	0.99782	
0.3 T1 vs 0.3 T3	0.80961	
0.3 T1 vs 0.05 T1	0.25151	
0.3 T1 vs 0.05 T2	1.00000	
0.3 T1 vs 0.05 T3	0.99987	
0.3 T1 vs 0.013 T1	0.00000	*
0.3 T1 vs 0.013 T2	0.00000	*
0.3 T1 vs 0.013 T3	0.00000	*
One-Sided T1 vs One-sided T2	1.00000	
One-Sided T1 vs One-Sided T3	0.92988	
One-Sided T1 vs Gentamicin T1	0.00000	*
One-Sided T1 vs Gentamicin T2	0.00000	*
One-Sided T1 vs Gentamicin T3	0.00000	*
One-Sided T1 vs 0.3 T2	0.63058	
One-Sided T1 vs 0.3 T3	1.00000	
One-Sided T1 vs 0.05 T1	0.99919	
One-Sided T1 vs 0.05 T2	0.94210	
One-Sided T1 vs 0.05 T3	0.99998	
One-Sided T1 vs 0.013 T1	0.00000	*
One-Sided T1 vs 0.013 T2	0.00000	*
One-Sided T1 vs 0.013 T3	0.00000	*
One-Sided T2 vs One-Sided T3	0.91920	
One-Sided T2 vs Gentamicin T1	0.00000	*
One-Sided T2 vs Gentamicin T2	0.00000	*
One-Sided T2 vs Gentamicin T3	0.00000	*
One-Sided T2 vs 0.3 T2	0.58079	
One-Sided T2 vs 0.3 T3	1.00000	

Continued on next page

Table 2 continued

Comparison Groups ($\frac{W}{cm^2}$)	P-value	Significance at $\alpha = 0.01$
One-Sided T2 vs 0.05 T1	0.99900	
One-Sided T2 vs 0.05 T2	0.92694	
One-Sided T2 vs 0.05 T3	0.99997	
One-Sided T2 vs 0.013 T1	0.00000	*
One-Sided T2 vs 0.013 T2	0.00000	*
One-Sided T2 vs 0.013 T3	0.00000	*
One-Sided T3 vs Gentamicin T1	0.00000	*
One-Sided T3 vs Gentamicin T2	0.00000	*
One-Sided T3 vs Gentamicin T3	0.00000	*
One-Sided T3 vs 0.3 T2	1.00000	
One-Sided T3 vs 0.3 T3	0.66916	
One-Sided T3 vs 0.05 T1	0.19429	
One-Sided T3 vs 0.05 T2	1.00000	
One-Sided T3 vs 0.05 T3	0.99358	
One-Sided T3 vs 0.013 T1	0.00000	*
One-Sided T3 vs 0.013 T2	0.00000	*
One-Sided T3 vs 0.013 T3	0.00000	*
Gentamicin T1 vs Gentamicin T2		
Gentamicin T1 vs Gentamicin T3	1.00000	
Gentamicin T1 vs 0.3 T2	0.00000	*
Gentamicin T1 vs 0.3 T3	0.00000	*
Gentamicin T1 vs 0.05 T1	0.00005	*
Gentamicin T1 vs 0.05 T2	0.00000	*
Gentamicin T1 vs 0.05 T3	0.00000	*
Gentamicin T1 vs 0.013 T1	1.00000	
Gentamicin T1 vs 0.013 T2	1.00000	
Gentamicin T1 vs 0.013 T3	1.00000	
Gentamicin T2 vs Gentamicin T3	1.00000	
Gentamicin T2 vs 0.3 T2	0.00000	*
Gentamicin T2 vs 0.3 T3	0.00000	*
Gentamicin T2 vs 0.05 T1	0.00000	*
Gentamicin T2 vs 0.05 T2	0.00000	*
Gentamicin T2 vs 0.05 T3	0.00000	*
Gentamicin T2 vs 0.013 T1	1.00000	
Gentamicin T2 vs 0.013 T2	1.00000	
Gentamicin T2 vs 0.013 T3	0.99997	
Gentamicin T3 vs 0.3 T2	0.00000	*

Continued on next page

Table 2 continued

Comparison Groups ($\frac{W}{cm^2}$)	P-value	Significance at $\alpha = 0.01$
Gentamicin T3 vs 0.3 T3	0.00000	*
Gentamicin T3 vs 0.05 T1	0.00000	*
Gentamicin T3 vs 0.05 T2	0.00000	*
Gentamicin T3 vs 0.05 T3	0.00000	*
Gentamicin T3 vs 0.013 T1	1.00000	
Gentamicin T3 vs 0.013 T2	1.00000	
Gentamicin T3 vs 0.013 T3	1.00000	
0.3 T2 vs 0.3 T3	0.24734	
0.3 T2 vs 0.05 T1	0.03718	
0.3 T2 vs 0.05 T2	1.00000	
0.3 T2 vs 0.05 T3	0.80983	
0.3 T2 vs 0.013 T1	0.00001	*
0.3 T2 vs 0.013 T2	0.00001	*
0.3 T2 vs 0.013 T3	0.00000	*
0.3 T3 vs 0.05 T1	0.99992	
0.3 T3 vs 0.05 T2	0.69213	
0.3 T3 vs 0.05 T3	0.99511	
0.3 T3 vs 0.013 T1	0.00000	*
0.3 T3 vs 0.013 T2	0.00000	*
0.3 T3 vs 0.013 T3	0.00000	*
0.05 T1 vs 0.05 T2	0.20294	
0.05 T1 vs 0.05 T3	0.69075	
0.05 T1 vs 0.013 T1	0.00000	*
0.05 T1 vs 0.013 T2	0.00000	*
0.05 T1 vs 0.013 T3	0.00000	*
0.05 T2 vs 0.05 T3	0.99584	
0.05 T2 vs 0.013 T1	0.00000	*
0.05 T2 vs 0.013 T2	0.00000	*
0.05 T2 vs 0.013 T3	0.00000	*
0.05 T3 vs 0.013 T1	0.00000	*
0.05 T3 vs 0.013 T2	0.00000	*
0.05 T3 vs 0.013 T3	0.00000	*
0.013 T1 vs 0.013 T2	1.00000	
0.013 T1 vs 0.013 T3	1.00000	
0.013 T2 vs 0.013 T3	1.00000	

Supplementary Table S3- ANOVA Analysis for Recoverability. Unpooled analysis amongst all trials to show significance at $\alpha = 0.01$. Data is log-normalized given analysis pre- sented in methods and results sections. T designates trial, astericks designates significance at given alpha level.

Comparison Groups ($\frac{W}{cm^2}$)	P-Value	Significance at $\alpha = 0.01$
0.3, t = 0 s vs 0.3, t = 60 s	0.00000	*
0.3, t = 0 s vs 0.3, t = 80 s	0.0000	*
0.3, t = 60 s vs 0.3, t = 80 s	0.9997	
0.3, t = 0 s vs 0.05. t = 0 s	0.9999	
0.3, t = 0 s vs 0.05, t = 60 s	0.0000	*
0.3, t = 0 s vs 0.05, t = 80 s	0.0000	*
0.3, t = 60 s vs 0.05, t = 0 s	0.0000	*
0.3, t = 60 s vs 0.05, t = 60 s	0.0000	*
0.3, t = 60 s vs 0.05, t = 80 s	0.0000	*
0.3, t = 80 s vs 0.05, t = 0 s	0.0000	*
0.3, t = 80 s vs 0.05, t = 60 s	0.0000	*
0.3, t = 80 s vs 0.05, t = 80 s	0.0000	*
0.05, t = 0 s vs 0.05, t = 60 s	0.0000	*
0.05, t = 0 s vs 0.05, t = 80 s	0.0000	*
0.05, t = 60 s vs 0.05, t = 80 s	0.0000	*

Supplementary Table S4 - Comparison of Stimulatory Methods. This table contains comparative information and metrics of various stimulatory techniques within the field, including kHz-MHz ultrasonics, electrical stimulation, and optogenetics.

Modality	Current Resolution & Toxicity Limits	Previous Work + Parameters	Effects	Limitations	Applications to Vagus Nerve Stimulation
KHz Ultrasound Stimulation	1 cm to m scale focal spot size in water, power levels of 1.9W/cm^2 are nontoxic, while 7.6 W/cm^2 is toxic (Lin et. al, 2012) [70]	Bachtold et al., 1998 (500kHZ carrier at repetition rate of 200kHz, 40-110 W/cm^2) [71] , Rinaldi et. al, 1991 (750 kHz, pulsed with 6 μs duration, average intensity of 80 W/cm^2) [72] , Gavrilov et. al, 1996 (480 kHz-3MHz, Pulsed, 1.3-30 kW/cm^2) [73]	Varied effects found in studies, as follows - No cavitation is observed and overall response is found to be a combination of ultrasonic and thermal effects, Depressed action potentials, Auditory and tactile stimulation	Lateral and Axial confinement are limited, effects are varying and challenging to target tissue of interest without invasive contrast agent delivery.	Vagus nerve inhibition and stimulation observed, potentially due to lack of localization to one fiber, one example study observes stimulation at 250 kHz, 3 W/cm^2 (Wasilczuk et. al, 2019) [74]

Continued on next page

Table 4 continued

Modality	Current Resolution & Toxicity Limits	Previous Work + Parameters	Effects	Limitations	Applications to Vagus Nerve Stimulation
MHz Ultrasound Stimulation	1 mm to tens of microns scale focal spot size in water, example efficacious to toxic threshold set at >1-3 W/cm^2 in varying studies, often for 1 minute or longer exposure times (Feril et. al, 2003 [75] ,Feng et. al, 2010 [76] , Ando et. al 2006 [77])	Colucci et. al, 2009 (600 kHz-2.7 MHz, Pulsed and CW, 1.5–18 kW/cm^2) [78] , Prieto et. al, 2017 (43 MHz, 50 or 90 W/cm^2) [79] , Muratore et. al, 2012 (4.04 MHz, 100ms pulses, calculated highest dose is 0.4 W/cm^{2}based on specified max pressure of 77 kPa) [80]	Varied effects found in studies, as follows - Decrease in conduction, Increase in stimulation of PIEZO-1, but not Na_V 1.2, Evoked potentials due to ultrasonic stimulus across different hippocampal regions	Axial resolution improved, but still not at single neuron, varying effects observed in terms of stimulation, coupled with potential delocalization from tissue of interest due to poor resolution	Vagus nerve inhibition and stimulation observed , potentially due to lack of localization to one fiber, example study notes primarily vagus nerve inhibition, at a frequency of 1.1 MHz, at intensities from 13.6-93.4 W/cm^2 (Juan et. al, 2014) [81]

Continued on next page

Table 4 continued

Modality	Current Resolution & Toxicity Limits	Previous Work + Parameters	Effects	Limitations	Applications to Vagus Nerve Stimulation
Optogenetics	λ of stimulus wavelength (100s nm), Light exposure < 50 mJ/cm^2 has been reported to be fully nontoxic for various cell lines (Maier et. al, 1991) [82]. Transfection toxicity is highly a function of methodology (Almulathanon et. al, 2018) [83].	Pegard et. al, 2017 (IR laser, 1 Hz pulsed, Computer holographics, 10-20 μm radius beam confinement) [84], Sun et. al (1.3 mm^2 field of view, 1.3 ms time frame, femtosecond laser pulsed stimulus) [85], Packer et. al, 2015 (Two photon stimulation of red-shifted opsin with cellular resolution) [86]	High resolution (micron scale) confinement of stimulus, Stimulation of cells with genetic modifications in field of view	Genetic transfection of cells presents toxicities, though high resolution, invasive probes needed for light to reach deep brain tissue, phototoxicities are present	Stimulation of individual afferent fibers observed in multiple studies, example studies use ChR2-YFP gene transfection of primary cortical E18 neurons, stimulated with pulsed 1 mW/mm^2, 455 nm light (Smith et. al, 2016) [87]

Continued on next page

Table 4 continued

Modality	Current Resolution & Toxicity Limits	Previous Work + Parameters	Effects	Limitations	Applications to Vagus Nerve Stimulation
Electrodes (Implantable or MEA)	Fabrication and impedance limits on electrodes leave smallest size at ~ 10 μm, focal size for stimulation larger depending on ground electrode location and sensing dependent on impedance of electrode, Gold, platinum and other inert metals are nontoxic (Merrill et. al, 2005) [88] , toxicities seen anywhere from 1-10 μC/ph (McCreery et. al, 1990) [89]	Sawahata, 2016 (5 μm needle implantable electrodes) [11] , Ronchi et. al 2019 (17.5 μm pitch, 70mV 100nA stimulation) [12]	Stimulation of action potentials in cells in contact with electrode array. Implantation efficiency and time course longevity variable and known to be limited within the field due to immune response	Confinement of stimulus is not only hard to measure but also strongly a function of device metrics, longevity due to immune response is known to be limited [13] [14] [15]	Stimulation of vagus nerve observed with various electrical stimulations via electrode interface, fine wire electrode is wrapped around fibers of interest – localization is physically manifested by wire placement, stimulus parameters for waveform are 20-30 Hz with a duration of 500 μs, and stimulation of 30-90 seconds (Johnson et. al 2018) [90]

Continued on next page

Table 4 continued

Modality	Current Resolution & Toxicity Limits	Previous Work + Parameters	Effects	Limitations	Applications to Vagus Nerve Stimulation
GHz Ultrasound Stimulation	1 μm and sub micron focal spot size possible. Single cell resolution achieved through imaging systems (Strohm et. al 2016, Khuir-Yakub et. al 1993) [22, 23]	Stimulation widely unreported in the literature apart from this study, however imaging parameter resolution has been investigated for different microscopy techniques. Routh et. al 1990 (Creation of 1 GHz differential phase acoustic lens with lateral resolution of 20 μm, potential for 20 Å scale phase resolution) [91] ,200-500 nm cavity depth achieved with visible object diameters of 300 nm for atomic force acoustic microscopy [92]	Outside of this study, characterization of stimulatory effects is lacking.	Penetration depth is limited for direct acoustic radiation pressure to tens of microns, although streaming has further possibilities to influence less localized structures.	No studies performed to date.

Continued on next page
